# Morphological, Molecular, and Biochemical Characterization of a Unique Lentil (*Lens culinaris* Medik.) Genotype Showing Seed-Coat Color Anomalies Due to Altered Anthocyanin Pathway

**DOI:** 10.3390/plants11141815

**Published:** 2022-07-10

**Authors:** Gyan P. Mishra, Ankita, Muraleedhar S. Aski, Mechiya Tomuilim Tontang, Priti Choudhary, Kuldeep Tripathi, Ajeet Singh, Ranjeet Ranjan Kumar, Vinutha Thimmegowda, Tsering Stobdan, Atul Kumar, Rakesh Bhardwaj, Shelly Praveen, Devendra Kumar Yadava, Shiv Kumar, Harsh Kumar Dikshit

**Affiliations:** 1 Division of Genetics, Indian Agricultural Research Institute, New Delhi 110012, India; ankita9876yadav@gmail.com (A.); murali2416@gmail.com (M.S.A.); mechiyalim965@gmail.com (M.T.T.); pritichaudhary.bu@gmail.com (P.C.); 2 Department of Biotechnology, Manav Rachna International Institute of Research and Studies, Faridabad 121004, India; 3 Germplasm Evaluation Division, National Bureau of Plant Genetic Resources, New Delhi 110012, India; kuldeep.tripathi@icar.gov.in (K.T.); rakesh.bhardwaj1@icar.gov.in (R.B.); 4 Division of Biochemistry, Indian Agricultural Research Institute, New Delhi 110012, India; dhakaajeet9961@gmail.com (A.S.); ranjeetranjaniari@gmail.com (R.R.K.); vinuthabiochem@gmail.com (V.T.); shellypraveen@hotmail.com (S.P.); 5 Defence Institute of High Altitude Research, Leh 194101, India; ts_mbb@yahoo.com; 6 Division of Seed Science and Technology, Indian Agricultural Research Institute, New Delhi 110012, India; atulpathiari@gmail.com; 7 Crop Science Division, Indian Council of Agricultural Research, New Delhi 110001, India; dkygenet@gmail.com; 8 South Asia and China Program, International Center for Agricultural Research in the Dry Areas, New Delhi 110012, India

**Keywords:** antioxidant activity, delphinidin, microgreens, sprouts, SSRs, testa color

## Abstract

This study reports the identification of a unique lentil (*Lens culinaris* Medik.) genotype L4717-NM, a natural mutant (NM) derived from a variety L4717, producing brown, black, and spotted seed-coat colored seeds in a single plant, generation after generation, in different frequencies. The genetic similarity of L4717 with that of L4717-NM expressing anomalous seed-coat color was established using 54 SSR markers. In addition, various biochemical parameters such as TPC (total phenolic content), TFC (total flavonoid content), DPPH (2,2-diphenyl-1-picrylhydrazyl), FRAP (ferric reducing antioxidant power), H_2_O_2_ (peroxide quantification), TCC (total carotenoids content), TAC (total anthocyanin content), and TAA (total ascorbic acid) were also studied in the seeds, sprouts, and seedlings of L4717, brown, black, and spotted seed-coat colored seeds. Stage-specific variations for the key biochemical parameters were recorded, and seedling stage was found the best for many parameters. Moreover, seeds with black seed coat showed better nutraceutical values for most of the studied traits. A highly significant (*p* ≤ 0.01) and positive correlation was observed between DPPH and TPC, TAA, TFC, etc., whereas, protein content showed a negative correlation with the other studied parameters. The seed coat is maternal tissue and we expect expression of seed-coat color as per the maternal genotype. However, such an anomalous seed-coat expression, which seems to probably be governed by some transposable element in the identified genotype, warrants more detailed studies involving exploitation of the anthocyanin pathway.

## 1. Introduction

Lentil is an important cool-season legume crop of India and is cultivated mainly as a rainfed crop [1]. Globally, it is cultivated in an area of 4.8 million ha and yields 5.73 Mt [2]. Canada (2.17 Mt) is the largest producer of lentils and is followed by India (1.23 Mt) and Australia (0.53 Mt). The market acceptability and value of lentils are very much dependent on seed traits such as testa color, testa pattern, and cotyledon color. Thus, to breed varieties having a specific testa color, a detailed understanding of the genetic mechanism controlling these traits is needed [3]. A very early report mentioning the monogenic nature of seed-coat color in lentil was presented by Tschermak Seysenegg [4], wherein he also observed a green seed coat (with purple spots) to be dominant over a light bland seed coat [5]. Erskine and Witcomb [6] identified five major classes of seed background color and three classes of seed-coat pattern in lentils. Monogenic expression of testa color with a dominance of brown color over tan and green was reported by Emami and Sharma [7]. Furthermore, cotyledon color was found to influence the testa color, which may be due to the diffusion of some soluble pigments from cotyledons to the testa. However, black testa color was found to be governed by two independent genes [8]. Similarly, the digenic inheritance of testa color was also reported by Vandenberg and Slinkard [9], while the testa pattern was found to be regulated by multiple alleles. In the case of cotyledon color, red was dominant over green and showed monogenic inheritance. Interestingly, green cotyledons showed the involvement of a recessive color inhibitor [10], and the *Dg* gene resulted in the formation of a dark-green cotyledon [11].

For the red lentil industry, efficient milling is one of the key economic traits; among various seed traits, seed-coat color is also known to influence milling efficiency [12]. Interestingly the seed-coat color and its composition are known to influence the seed-coat thickness. Genotypes with no tannins in their seeds (homozygous tan) show much lower polyphenol content [13]. Such seed coats are nearly 20% thinner and also nearly 20% lighter in weight than the genotypes having normal seed-coat color [14]. Two independently located genes (*Ggc* and *Tgc*) are known to determine the seed-coat color in lentils. Dominant and recessive combinations of these alleles at each locus determine the four basic seed-coat colors, namely, brown (*Ggc Tgc*), gray (*Ggc tgc*), tan (*ggc Tgc*), and green (*ggc tgc*). Differences in the contents (amount and type) of polyphenolic compounds in the seed coat ultimately determine the color of lentils. Higher contents of flavan-3-ols, proanthocyanidins, and certain flavones result in gray and green seed-coat color. Dark or red-colored genotypes of barley, sorghum, millet, and rye are colored due to the presence of anthocyanin compounds, while the reddish/brown color of red wheat, rice, and maize is due to oxidized proanthocyanidins [15,16,17,18,19]. In purple maize kernel, pigments are mainly concentrated in the external part of the seed (pericarp), while pigments in the aleurone layers of some species, such as blue maize kernel, have a colorless pericarp [20,21,22].

Generally, most of the colored seed-coat genotypes contain proanthocyanidins; however, all may not contain anthocyanins. On the other hand, black rice contains no or very small amounts of proanthocyanidins [18]. This is due to the activity of polyphenol oxidase, as well as the innate genetic differences present in the genotypes expressing anthocyanin reductase and/or leucoanthocyanin reductase. Anthocyanin reductase converts the anthocyanins into flavan-3-ols, which consequently can be polymerized into proanthocyanidins, whereas leucoanthocyanin reductase transforms leucoanthocyanins into flavan-3-ols, which subsequently polymerize into proanthocyanidins [23,24,25]. In both cases, proanthocyanidins after oxidation result in the formation of reddish/brown compounds. In a number of cereals, purple, blue, or black colors are due to the presence of anthocyanins [18,26,27,28]. A similar process was also recorded in legumes such as broad bean [29] and soybean [30].

In lentils, polyphenol oxidase is known to oxidize the colorless proanthocyanidins into colored compounds, while it also results in structural changes in the seeds [31]. It is the varying contents of various polyphenolic compounds which ultimately affect the dehulling process and, in turn, cause differing levels of loosening of seed coats from the cotyledons during milling. Seed parameter uniformity in lentils is essential for the marketing and preparation of various food products. Thus, seed-coat color variations in lentils have become a major research concern as these are reportedly affected by genetic, pathological, and various environmental factors [32]. Seed-coat background colors in lentils are an important trait for both growers and consumers. Multicolored lentil grains fetch lower prices over single- and uniform-colored lentils. Across the world, the preference varies for various types of lentils, such as uniform white, green, gray, brown, red, or black. Consumers in the United States and Europe prefer light, uniformly colored lentils [33], while Indian consumers prefer lentils with a dark seed coat [34]. 

Today, consumers are also becoming more health cautious and want nutritionally rich or biofortified lentil varieties. Black seed-coat lentils are a candidate for such consumers if thoroughly investigated for their nutraceutical qualities. Proanthocyanidins are the major group of polyphenols concentrated in the seed coats of lentils. The presence of high phenolic content in the lentil seed coat is desired for both the food and the pharmaceutical industries primarily as a source of natural antioxidants [35]. Therefore, breeders must know the genes or QTLs regulating the seed-coat color formation in lentils. This will help in the development of customized seed-coat colored varieties in lentils [33]. With this background, this study was aimed at characterizing a unique lentil genotype having an anomalous expression of seed-coat color (brown, spotted, and black in a single plant), along with its parent (L4717), at the morphological, molecular, and biochemical levels at different growth stages (seeds, sprouts, and seedlings).

## 2. Results

### 2.1. Seed-Coat Color Anomalies

Development of L4717 began in the year 2011–2012 wherein a nursery derived from the cross ILL7617 × 91516 was received from ICARDA, Lebanon for its further evaluation in India. It is interesting to mention that the progenies of this nursery always produced some black seeds (<1%) during seed production since the development of this variety in the year 2011–12 until now. Each year before sowing, we manually removed the black seeds and then performed sowing. However, to our surprise, we always found some black seeds (<1%) in the final seed lot. 

**Figure 1:**
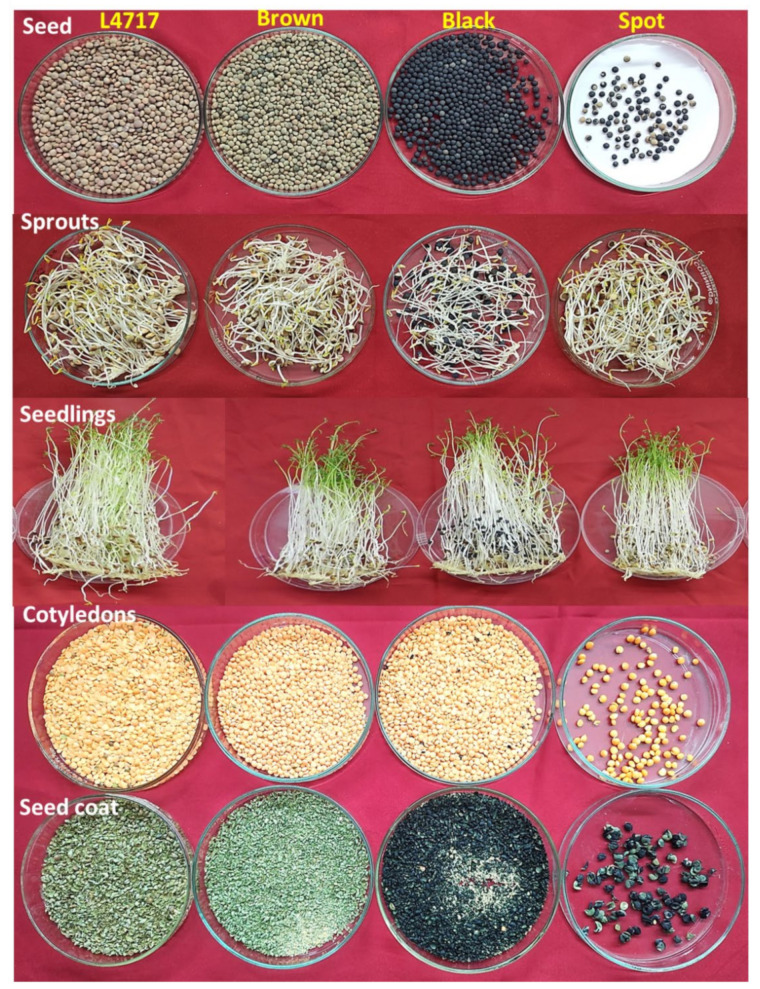
Phenotypic details of seeds, sprouts, seedlings, cotyledons, and seed coats of the genotypes L4717 and L4717-NM (brown, black, and spotted seed-coat types).

In the year 2017–2018, we selected one plant (L4717-NM) which, when threshed, showed a mixed type of seed-coat expression having brown (98), black (29), and spotted seeds (10). Such an anomalous seed-coat color expression was unexpected and motivated a single-plant analysis. The seeds were again sown in the years 2018–2019, 2019–2020, and 2020–2021, and data were recorded for the seed-coat color (Table 1). To avoid any kind of chance cross-pollination, the flower buds were covered with small cotton balls of three plants in each category. Interestingly, brown, black, and spotted seeds produced all three types of progenies having brown, black, and/or spotted seeds. Some of the brown, black, and mixed genotypes produced a few seeds having spotted seed-coat patterns. The detailed plant data are presented in Table 2. The plants of L4717-NM look morphologically very similar to those of L4717. Moreover, the cotyledons are red irrespective of their seed-coat color (Figure 1). The L4717-NM pods having black seeds possess anthocyanin pigmentation only at the time of seed development (before maturity), after which they become faded as pods fully mature. 

**Figure 2:**
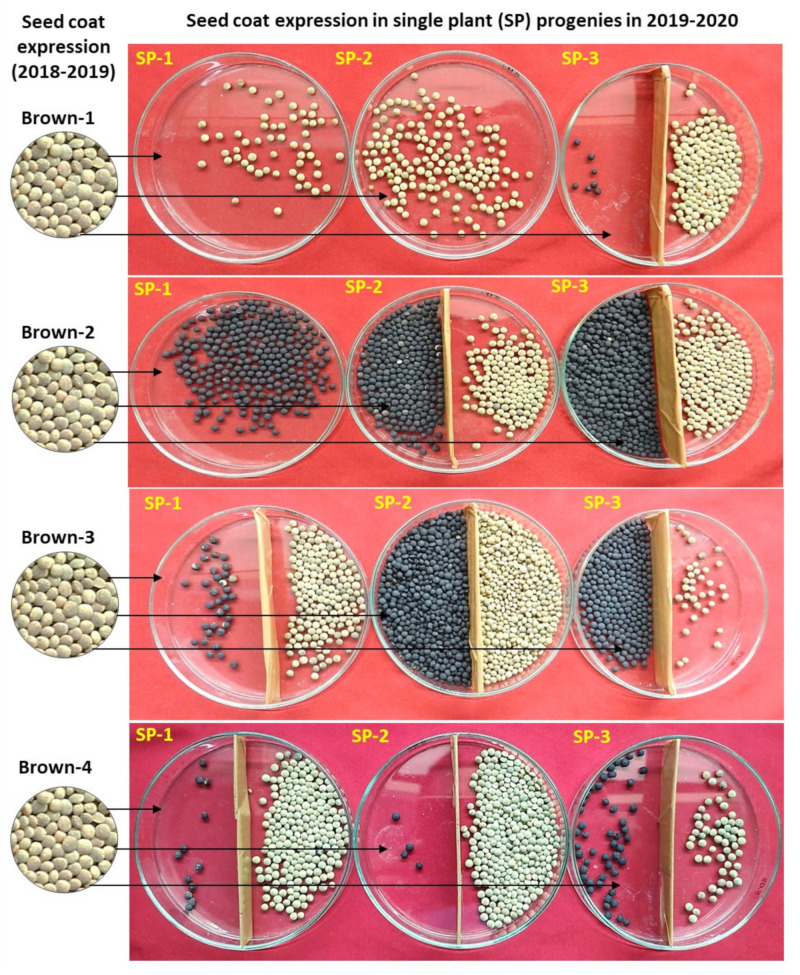
Seed-coat expression of three single plants (SP-1 to SP-3) in the year 2019–2020 derived from the four plants seeds (L4717-NM) having brown seed-coat expression (Brown-1 to Brown-4) in the year 2018–2019. Black/spotted and brown seed-coat colored seeds (if formed) were separated in a Petri plate (obtained from a single plant) for more clarity of their relative frequencies per plant.

**Figure 3:**
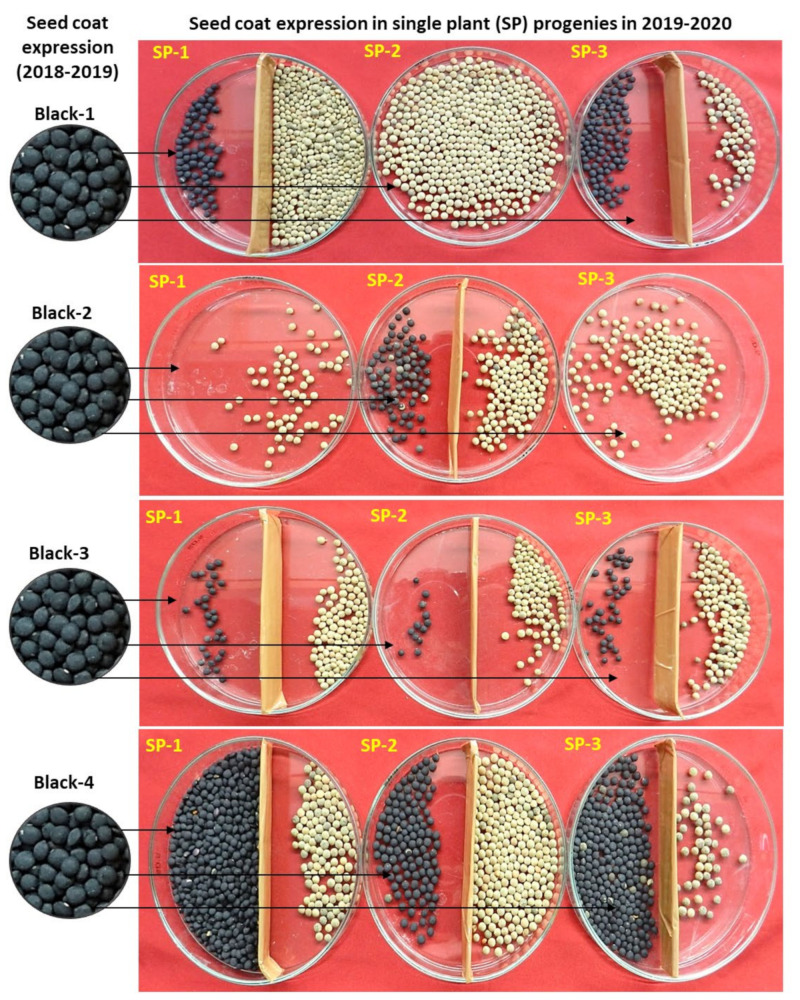
Seed-coat expression of three single plants (SP-1 to SP-3) in the year 2019–2020 derived from the four plants seeds (L4717-NM) having black seed-coat expression (Black-1 to Black-4) in the year 2018–2019. Black/spotted and brown seed-coat colored seeds (if formed) were separated in a Petri plate (obtained from a single plant) for more clarity of their relative frequencies per plant.

**Figure 4:**
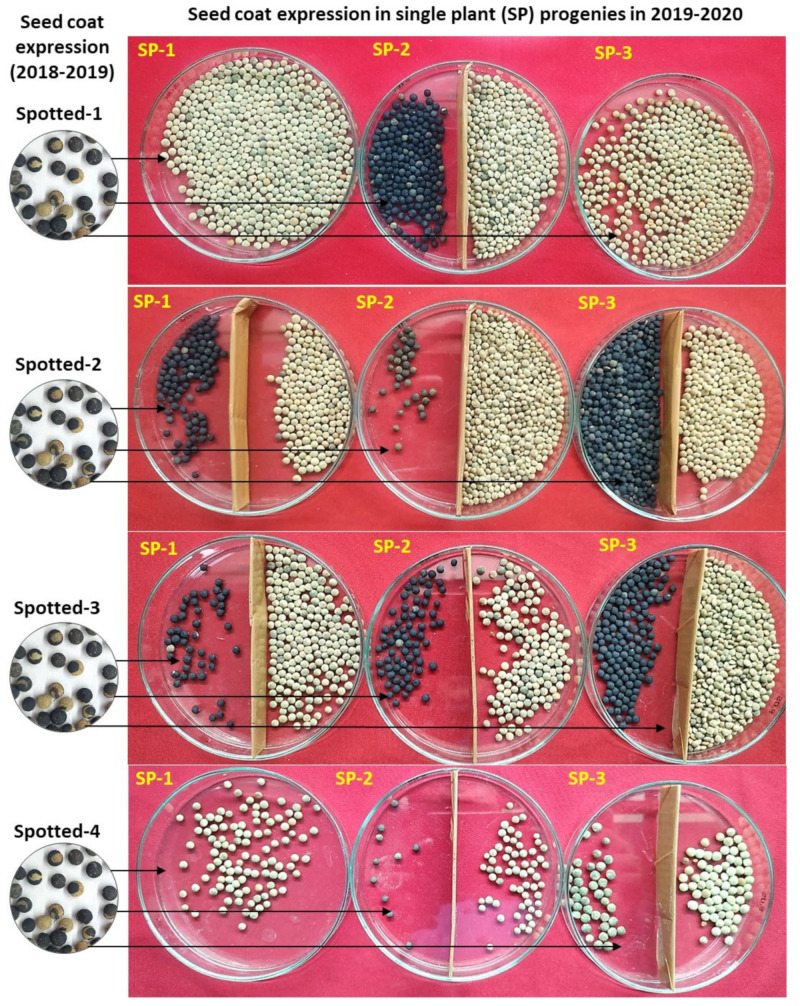
Seed-coat expression of three single plants (SP-1 to SP-3) in the year 2019–2020 derived from the four plants seeds (L4717-NM) having spotted seed-coat expression (Spotted-1 to Spotted-4) in the year 2018–2019. Black/spotted and brown seed-coat colored seeds (if formed) were separated in a Petri plate (obtained from a single plant) for more clarity of their relative frequencies per plant.

From the 3-year data, it is very clear that the plants producing mixed (brown, black, and spotted) types of seeds formed all three categories of plants (brown, black, and mixed in different frequencies). Moreover, when brown, black, and spotted seed-coat colored seeds were separately sown in different rows, they produced all three types of progenies (black, brown, and mixed). Overall, the number of plants producing only black seed-coated seeds was much lower compared to brown or mixed type. Interestingly, no trend was recorded between the seed-coat color of the parent and the progenies (Figure 2, Figure 3 and Figure 4). This could be the reason why we are unable to completely purify the L4717 seed lot, as the brown-looking seeds produce black and spotted seed-coat colored seeds in the next generation (although in very low frequency).

To gain more clarity about the anomalous seed-coat color expression, we also analyzed the progenies derived from the brown, black, and spotted seed-coat seeds (Table 2, Figure 2, Figure 3 and Figure 4). Interestingly, plants derived from the brown, black, and spotted seeds (2018–2019) produced all three types of progenies (black, brown, and mixed) in 2019–2020. In addition, the ratio of brown, black, and spotted seeds varied in different plant progenies. Again, no specific trend could be established in the seed-coat color of the progenies when compared with that of parental seed-coat color.

### 2.2. Molecular Marker Analysis

To confirm the genetic similarity or dissimilarity of the identified genotype (L4717-NM) with that of the L4717, molecular analysis was performed using SSR primers. A total of 54 SSR primer pairs were used for the amplification of four genotypes (based on seed-coat color expression), which included (1) L4717- and L4717-NM-derived seeds, (2) brown seed-coat seeds, (3) black seed-coat seeds, and (4) spotted seed-coat seeds. As mentioned earlier, the seeds of L4717-NM were obtained from a single plant which was selected in the year 2017–2018. All the amplified primers showed monomorphic expression (Figure S1) which confirmed the genetic similarity of L4717-NM with that of L4717 even at the DNA level.

### 2.3. Biochemical Studies

Biochemical studies including the estimation of TPC, TFC, DPPH scavenging, FRAP, peroxide quantification, TCC, crude protein, TAC, and TAA were performed for four distinct lentil genotypes (based on the seed-coat color) including L4717 at three stages, namely, seeds, sprouts (5 days), and seedlings or microgreens (11 days). Significant variations were recorded for most of the studied biochemical traits, especially for the growth stages. The TPC was recorded ranging from 1047.64 to 2434.27 (mg GAE/100g DW) with a maximum in the seedlings derived from black seed-coat seeds and a minimum in spotted seed-coat seeds. The range of other biochemical parameters such as TFC (1.91 to 3.28 mg/g DW), FRAP (7.35 to 46.04 µmol Fe^2+^/g DW), TCC (2.55 to 45.35 mg/100 g DW), TAA (27.21 to 44.12 mg/100 g), and DPPH scavenging activity (35.97 to 66.46%) also varied across different growth stages. Peroxide content was recorded to be maximum in brown seed-coat sprouts (2.95 nmol/g DW) and minimum in L4717 seeds (1.64 nmol/g DW). As expected, crude protein was found to be maximum in the seeds (26.43 g/100 g DW), followed by the sprouts, and minimum in the seedlings (9.52 g/100 g DW) of the studied genotypes. The TAC was recorded as maximum in black seed-coat seeds (0.042 mg/g DW as delphinidin equivalent), followed by spotted seed-coat seeds (0.033 mg/g DW), and minimum in L4717 seeds (0.016 mg/g DW). Interestingly, TAC was not recorded either in the sprouts or in the seedlings. Overall, TPC, TFC, DPPH, FRAP, H_2_O_2_, and TAA were found to be maximum in the seedlings and minimum in the seeds of the studied genotypes. TCC was recorded to be maximum in the sprouts and minimum in the lentil seeds. However, protein was recorded to be maximum in the seeds and minimum in the seedlings. The genotype-based details of all parameters are presented in Table 3.

### 2.4. Correlation Analysis

**Figure 5:**
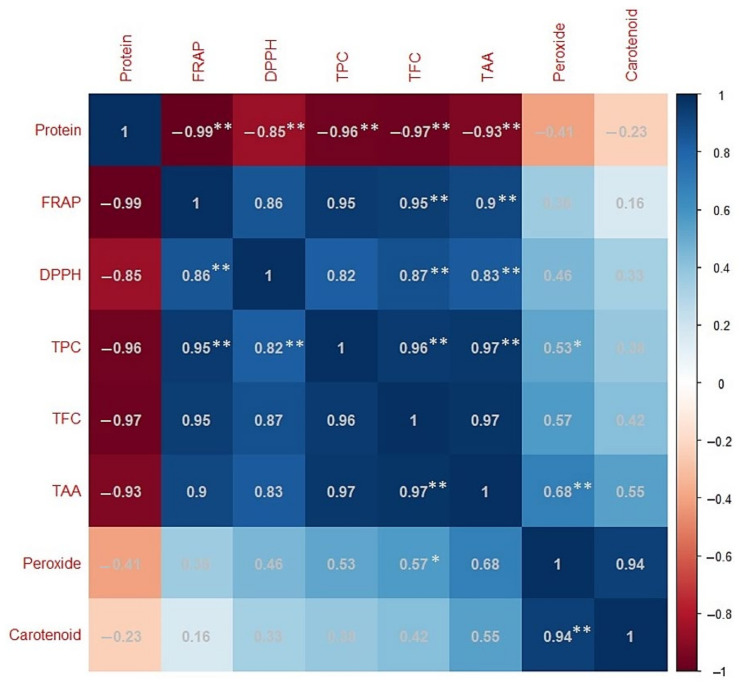
Correlation matrix of results obtained from data on eight metabolites for lentil samples differing in terms of seed-coat color. Each square indicates the Pearson’s correlation coefficient for a pair of metabolites, and the value for the correlation coefficient is represented by the intensity of the blue or red color as indicated on the color scale. Correlation is significant at the (**) *p* ≤ 0.01 and (*) *p* ≤ 0.05 levels.

The correlation between measured values of various biochemical parameters (TPC, TFC, DPPH, FRAP, peroxide, TCC, crude protein, and TAA) was estimated using Pearson’s correlation method (Figure 5). A highly significant (*p* ≤ 0.01) and positive correlation was recorded between DPPH and FRAP (*r* = 0.8611 **), TPC (*r* = 0.8238 **), TAA (*r* = 0.8332 **), and TFC (*r* = 0.8741 **). Similarly, FRAP activity also showed a highly significant (*p* ≤ 0.01) and positive correlation with TPC (*r* = 0.9534 **), TAA (*r* = 0.9067 **), and TFC (*r* = 0.9517 **). Interestingly, protein content showed a negative correlation with all studied parameters; a highly significant (*p* ≤ 0.01) and negative correlation was recorded with DPPH (*r* = −0.8531 **), FRAP (*r* = −0.9904 **), TPC (*r* = −0.9602 **), TAA (*r* = −0.9291 **), and TFC (*r* = −0.9704 **). It is noticeable that TCC did not show a significant correlation with any of the studied parameters, whereas peroxide activity showed a highly significant correlation with TCC (*r* = 0.9390 **). Other details are presented in Figure 5. 

### 2.5. Cluster and PCA Analysis of Functional Features of Lentil Genotypes

**Figure 6:**
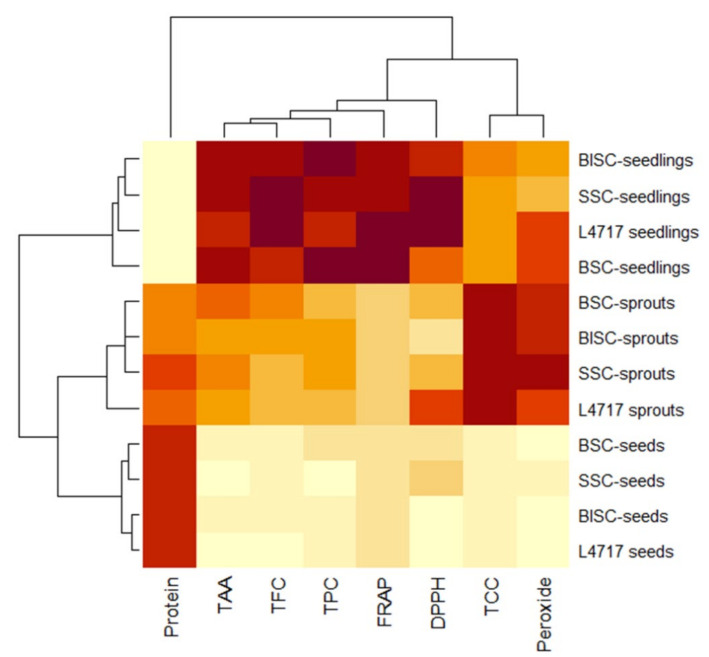
Hierarchical cluster analysis and heatmap visualization of physiochemical, nutritional, and nutraceutical data of 12 lentil samples. Diverse colors in each row indicate differences in the values estimated for each parameter among the 11 samples. BSC (brown seed coat), BlSC (black seed coat), and SSC (spotted seed coat) from L4717-NM genotype.

**Figure 7:**
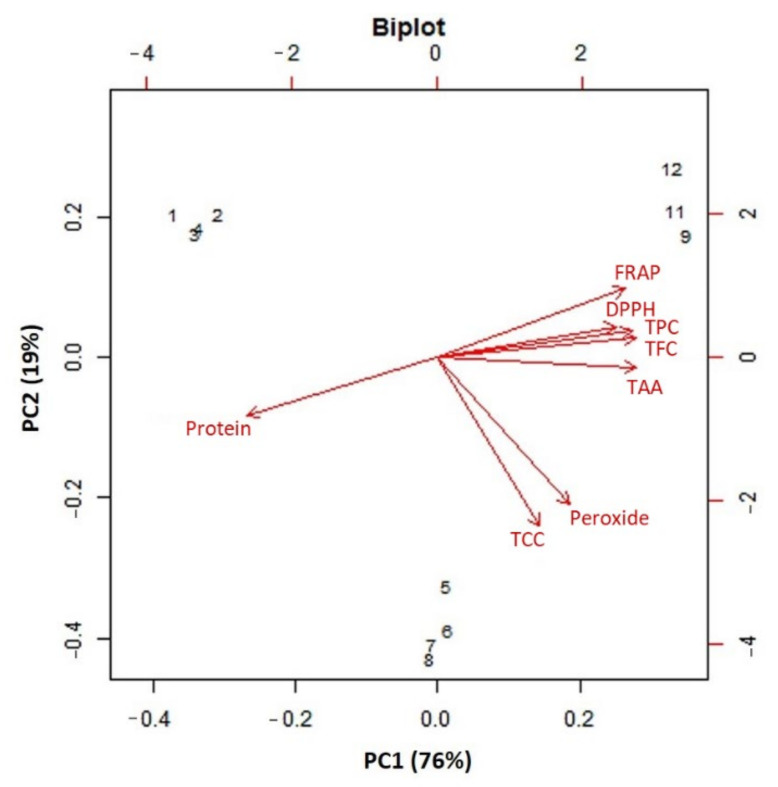
Principal component plot derived from various antioxidant activities [(1,1-diphenyl-2-picrylhydrazyl (DPPH), ferric-reducing antioxidant power (FRAP), peroxide, total carotenoid content (TCC), and total ascorbic acid (TAA)], protein, TPC, and TFC in the lentil genotypes.

Hierarchical cluster analysis and heatmap visualization of physiochemical (DPPH, FRAP, and peroxide), nutritional (protein), and nutraceutical data (TAA, TCC, and TFC) of 12 lentil samples were performed. The results showed that the lentil genotypes could be grouped into two major clusters. Interestingly, the seeds, sprouts, and seedlings were clearly grouped into different clusters or subclusters (Figure 6). The AoA changed significantly, from seeds to sprouts and then to microgreens; this was seemingly the reason for the clear grouping of the genotypes into very distinct stage-specific clusters. The clustering also showed that, within the studied genotype, there was not much significant difference at any particular growth stage. Furthermore, the functional characteristics such as AoA, protein, and polyphenols of lentil genotypes having different seed-coat colors were used to perform the PCA (Figure 7). The PCA showed that the first two principal components (PCs) explained very high variance (95%), wherein PC1 and PC2 explained 76% and 19% of the total variance, respectively.

## 3. Discussion

The improvement of seed morphology associated with economic traits such as seed-coat color in legumes needs an oriented exploitation of existing genetic diversity [36]. The development of a lentil genotype having desirable seed-coat color needs a proper understanding of the genetic basis of that trait, which will help in the acceptance of such varieties by different market segments, as well as in fetching a better price. This study aimed to characterize a lentil genotype having anomalous seed-coat color expression at morphological, molecular, and biochemical levels. It is essential to mention that we observed the presence of black and spotted seeds in the L4717 seed lot since the very beginning when we received the parental nursery of this genotype from ICARDA in the year 2011–2012. Each year, we manually removed the black and spotted seeds from the seed lot before sowing. However, up until 2021, we were unable to purify our L4717 seed lot, with <1% seeds still having either black or spotted seed-coat color.

### 3.1. Morphological Characterization

In general, the seed-coat ground color and pattern are reportedly under the control of dominance and codominance effects, while a black seed coat is independent of any true ground color [9]. In contrast, this study reports the formation of black, brown, and spotted seed-coat colored seeds in a single lentil plant, generation after generation. Interestingly Wilson and Hudson [32] reported the expression of black, mottled, and bicolored seed coat in early generations (F_2_ and F_3_) from a cross derived from black and beige seed-coat colored parents. However, our study reports such seed-coat expression in a fixed genotype generation after generation without any specific pattern. Mottled, bicolor, or spotted patterns were observed on lentil seed backgrounds ranging from black to brown. Furthermore, a range of color patterns were recorded at a single-plant basis at random frequencies, as also observed by Wilson and Hudson [32]. Early generation (F_1_) seed-coat mottling has also been reported in other pulses such as *Phaseolus vulgaris* [37] and in *Vigna sinensis* (L.) Savie × Hassk [38]. Fruwirth [39], while studying the lentil seed-coat color expression for eight generations in a genotype ‘Krain’ having partly solid brown and partly black spotted seeds, recorded the appearance of both kinds of seeds on the same plant. Interestingly, he also could not succeed in getting the black spots to full heredity, and even the selection of plants with no spotted seeds failed to produce a line having unspotted seeds. A similar observation was also recorded for other crops such as vetch (*Vicia sativa*), snap beans (*Phaseolus vulgaris*), fodder pea (*Pisutn arvense*), and white mustard (*Sinapis alba*), as well as in another lentil genotype ‘Puy’ after rounds of selfing and selections [39]. Such an anomalous seed-coat color in the cases of mottled beans and spotted peas was also observed and termed as a pseudo-mutation by Imai [40] (1935). Even for the chlorina and variegated mutants of okra (*Abelmoschus esculentus*), an unstable gene was reported [41]. Interestingly, in previous studies, researchers did not study the mottling pattern, especially in later generations using seed-coat color-based planting and a plant-by-plant study. Our study signifies the importance that detailed studies are needed for the expression of seed-coat color especially in the later generations (of certain crosses) using plant-by-plant and pod-by-pod approaches in lentils and other pulses. This will certainly help in reconfirming the presence of such a phenomenon in other pulses and will open a new frontier for unfolding the exact reason(s) behind such an anomalous expression of the seed-coat color in a few genotypes. 

Punia et al. [42] obtained a natural mutant plant from an Indian variety DPL-62 in the year 2007–2008, which showed variation for earliness and other morphological traits including seed-coat color. They also reported that the progenies of this mutant plant were segregated until the sixth generation of its identification. Due to such an abundance of the variations in the studied traits, these identified genotypes are named hypervariable spontaneously generated plants (HVSGPs) and are considered the source of new alleles. In contrast, in our study, the anomalous expression of seed-coat color is not limited to a single plant and has persisted in the population since we received the genotype as a nursery from ICARDA, Lebanon in the year 2011–2012. In addition, we studied this anomalous seed-coat color expression in the identified genotype (L4717-NM) on a single-plant and pod-to-pod basis from 2017–2018 until 2020–2021. Furthermore, except for the seed-coat color, L4717-NM is the same as its parental genotype L4717. Thus, a few reports of anomalous expression of several traits have been reported in lentil [42], but such an anomalous seed coat-color expression has never been reported in lentil or in any other crop.

### 3.2. Molecular Characterization

Molecular analysis was performed for L4717 and L4717-NM (brown, black, and spotted seeds) using 54 SSR primers, which showed a monomorphic banding pattern for all SSR primer pairs in the studied genotypes. This again confirmed the genetic similarity of L4717-NM with that of L4717, from which this genotype was identified. In addition, L4717 is a very early maturing variety that matures under Delhi conditions in nearly 100–102 days. Interestingly, the maturity duration and other morphological features of the identified genotype are also the same, which again confirms that L4717-NM is derived from L4717. Otherwise, lentil is a naturally highly self-pollinated crop having cleistogamous flowers [43,44], and, with <0.8% natural cross-pollination [45], the possibility of intragenic recombination is negligible [42]. In soybean, the seed color (black, brown and brown striped) was found to be regulated via *w4* and *R* loci [46,47], and methylation of the *R* locus was found to affect the transposition and splicing of a large CACTA transposon from an MYB transcription factor regulating anthocyanin synthase genes [48]. We may expect such epigenetic factors regulating seed-coat color expression in the identified lentil genotype, which can be tested in future studies using this genotype.

### 3.3. Biochemical Characterization

Various antioxidant assays including anthocyanin estimation and total crude protein were performed in four lentil samples differing in terms of seed-coat color to identify the significant differences (if any) on the basis of genotype and growth stages. Different AoA-associated mechanisms can be exploited by determining the varied modes of action such as reducing ability (FRAP), free-radical-scavenging ability (DPPH), preventive effect against lipid peroxidation (H_2_O_2_), and ability to chelate transition metals ions [49]. Normally, flavonoids possess more AoA than phenolics [50] and play a key role in the cell-division-related signal transduction pathways, thereby imparting anticancer properties [51]. Interestingly, a significant stage-wise increase was recorded for TPC (1047.64 to 2434.27 mg GAE/100 g DW) and TFC (01.91 to 3.28 mg/g DW) when studied in the lentil samples (differing in terms of seed-coat color) at different stages (seeds, sprouts, and seedlings). Similarly, in the 6-day old lentil sprouts, nearly similar TPC (19.4 mg/g DW) was reported by Swieca et al. [52]. Furthermore, in the lentil seeds, Fouad et al. [53] and Altuner [54] reported TPC to the tune of 1510.10 and 385.44 mg/100 g DW, respectively. A similar range of TPC was also reported for mustard (1889.76 mg/100 g FW) and radish (211.19 mg/100 g FW) seedlings [55]. Additionally, in lentil microgreens (seedlings), a similar range of TPC (212.60 to 321.35 mg GAE/100 g FW) was reported [56]. 

Nearly similar TFC was also reported in 4 day (1.71 mg QE/g DW) and 6 day (1.68 mg QE/g DW) old lentil sprouts [52]. Moreover, TFC to the tune of 4.96 mg catechin/g DW [53] and 0.57 mg/g DW [57] was reported in 6-day old lentil sprouts, while, in 20-day old lentil seedlings, this was recorded as 2.77 mg QE/g [53]. Interestingly, a significant stage-wise increase was recorded for DPPH scavenging (35.97 to 66.46%), FRAP (7.35 to 46.04 µmol Fe^2+^/g DW), and TAA (27.21 to 44.12 mg/100 g), when studied for the lentil samples (differing in terms of seed-coat color). A similar range of total AoA was recorded (DPPH: 0.84–3.38 µmol TE/g FW; FRAP: 14.7–55 µmol TE/g FW) in lentil microgreens [56]. Furthermore, Fouad et al. [53] reported 40.76% DPPH scavenging activity in seeds and 62.19% activity in 6-day old lentil sprouts, whereas Swieca et al. [52] reported 0.32 mmol TE/g DW of DPPH inhibition in 6-day old lentil sprouts. Ahuja et al. [58] reported FRAP scavenging activity as 0.25–7.25 µmol Fe^2+^/g DW in the lentil genotypes, while Wojdylo et al. [59] recorded 0.1 mmol TE/100 g FW of FRAP activity in the lentil sprouts.

H_2_O_2_ in leaf tissue is known to respond to various environmental stimuli [60]. Hydrogen peroxide acts as a signaling molecule in the plant system and imparts a second line of defense; an increased level of H_2_O_2_ is associated with cellular oxidative damage [61]. Interestingly, the H_2_O_2_ was recorded to be the minimum (1.64 nmol/g DW) in the seeds, followed by seedlings, and the maximum (3.04 nmol/g DW) in the sprouts. A similar range was also recorded for microgreens (1.63–5.92 nmol/g FW) by Priti et al. [56]. In seven lentil genotypes, the peroxide activity was reported in the range of 1.22–2.22 µmol/g [62]. The conjugated double bonds in the carotenoid result in AoA via free-radical scavenging [63]. In our study, TCC (2.55 to 45.35 mg/100 g DW) was recorded to be minimum in the seeds, followed by the seedlings, and maximum in the sprouts. Seedlings of the black genotype showed the highest value among the 12 samples studied. A similar range of carotenoids was reported in the seeds of red lentil (5.31–28.1 µg/g DW) and green lentil cultivars (6.20–16.4 µg/g DW) [64]. Additionally, in peas (5.41–28.19 µg/g DW) and chickpea seeds (8.86–30 µg/g DW), nearly similar rage were recorded for TCC [65]. Interestingly, in lentil microgreens, a similar range of TCC was recorded. i.e., 28.37 mg/100 g FW [66] and 14.40–22.22 mg/100 g FW [56]. However, in lentil sprouts, Wojdylo et al. [59] also reported a similar TCC (6.4 mg/100 g FW).

Our study recorded the maximum protein in seeds (26.43 g/100 g DW), followed by sprouts, and the minimum in microgreens (9.52 g/100 DW). Similarly, seed protein content was recorded as 25.63 g/100 g DW [53], while in 4- and 6-day sprouts, the protein level was 22.7 g/100 g DW and 22.1 g/100 g DW, respectively [67]. In microgreens, the protein level was 1.84–2.67 mg/100 g FW [56]. Fouad et al. [53] reported more free amino acids in the sprouts (13.80 mg/g DW) than in the lentil seeds (1.86 mg/g DW). Thus, the reduction in the total protein content from seeds to seedlings is due to the breaking down of proteins into free amino acids as required for the growth and development of lentil seedlings.

As recorded in this study, nearly similar TAA (289.2 µg/g DW) was also reported in 4-day old lentil sprouts [67] and in microgreens (16.15–26.86 mg/100 g FW) of lentils [56]. Since ascorbic acid is present mostly in chloroplasts, the increased value in seedlings is due to the formation of chloroplasts [68,69]. In addition, vitamin C content is usually high in photosynthetic tissues but low in non-photosynthetic tissues. Some orthodox categories of seeds, which reach maturity in a state of strong dehydration, contain little vitamin C [69]. A similar trend of TAA was recorded in this study, wherein the maximum TAA was recorded in seedlings, followed by sprouts and seeds.

### 3.4. Correlation, Cluster and PCA Analysis

The AoA in the studied lentil samples was found to be linearly correlated according to the FRAP and DPPH methods. On a similar note, Priti et al. [56] also reported a positive correlation between DPPH and TFC (*p* ≤ 0.01) in lentil microgreens and between FRAP and DPPH activity (*p* ≤ 0.01) in mungbean microgreens. In sweet potatoes, a significant positive correlation between DPPH and FRAP (*r* = 0.966) was also recorded [70]. This means that the phenolic and flavonoid compounds mainly contribute to the overall AoA in the studied lentil genotypes. Such observations were also reported for *Capparis* leaves [71,72]. Similarly, FRAP was found to be significantly correlated with TAA (*p* ≤ 0.05) in lentil microgreens [56] indicating their role in the AoA of lentil seeds, sprouts, and microgreens. Thus, correlation studies have provided a clear understanding of the parameter(s) contributing to the total AoA [73,74,75]. Proteins are the main energy source for lentil seeds, and the negative correlation between total protein content and all other studied parameters indicates that the protein is being used to produce various types of AoA in the sprouts and seedlings. 

Cluster analysis distinctly grouped the lentil genotypes according to their growth stages (seeds, sprouts, and seedlings). A similar kind of clustering pattern was observed for the AoA in lentil and mungbean genotypes when studied at the seedling stage [56]. Furthermore, for different caper (leaves) genotypes when collected from different parts of Ladakh, a similar kind of genotypic clustering was recorded for the total AoA [71]. In plants, phenolics are well reported to have a key role in their total bioactive activities [76]; thus, a positive association between phenols and the total AoA is a common trend in many crop species [77].

PCA is useful for understanding the variations at the species level across different attributes in response to diverse factors such as genetic makeup, maturity duration, and growing conditions [78,79,80]. PCA showed that 95% of the variation was explained by the first two PCs in the studied lentil samples. Similarly, for the lentil and mungbean seedlings, when grown under Delhi conditions, the first two PCs explained 43.68% and 52.7% of the total variance, respectively [56]. Moreover, for lettuce seedlings, the first two PCs explained 99.1% of the total variance [81]. 

### 3.5. Probable Effect on the Anthocyanin Biosynthesis Pathway

Seed-coat pigmentation in pulses happens through the deposition of various flavonoids which are mainly derived from the anthocyanin biosynthesis branch of the phenylpropanoid pathway [82]. Anthocyanins are water-soluble colored pigments having health benefits due to their antioxidative and antimicrobial activities [83]. The presence of a number of mutants affecting the expression of several anthocyanin biosynthetic pathway genes altering seed pigmentation in a number of crops such as maize, petunia, snapdragon, and gerbera has facilitated the cloning of various regulators [84]. Black seed-coat lentils are known to have delphinidin-3-glucoside as the major anthocyanin component [85]. In our study, TAC was (estimated as delphinidin equivalent) recorded in the range of 0.016 (L4717) to 0.042 mg/g DW (black seed-coat seeds). A nearly similar range of anthocyanins was reported in green lentil (0.018 mg/g DW) and red lentil (0.02 mg/g DW) seeds [86]. Interestingly, anthocyanin could not be detected in sprouts or microgreens/seedlings. Similarly, the nutritional profiling of 14 different microgreens species including lentils reported the presence of anthocyanins only in purple radish and red cabbage [66]. As expected, significantly more TAC was recorded in black and spotted seed-coat seeds than in L4717 or brown seed-coat seeds. This means that the anthocyanin pathway is more active in the black and spotted seed-coat seeds compared to brown seed-coat seeds (even in the same plant). It is also very clear that the flavonoid pathway is affected very easily and frequently, even in the same plant of L4717-NM, by certain unknown factors, thereby resulting in the anomalous seed-coat color expression. 

In the flavonoid pathway, there are two major groups of genes: early biosynthetic genes (CHS: chalcone synthase, CHI: chalcone isomerase, F3’H: flavonoid 3′-hydroxylase, and F3′5′H: flavonoid 3′,5′-hydroxylase) and late biosynthetic genes (DFR: dihydroflavanol reductase and AS: anthocyanidin synthase). EBGs are associated with the formation of common precursors (dihydroflavonols) [87]. The corresponding genes are associated with the formation of seed-coat pigment, and mutation in these genes may result in transparent testa as reported in *Arabidopsis* [88]. Furthermore, in Chinese cabbage, whole-genome sequencing identified TRANSPARENT TESTA GLABRA 1 (TTG1) as the likely candidate gene controlling seed-coat color [89]. Similarly, in soybean, the R and T loci are known to control various seed-coat colors including black (i, R, T), imperfect black (i, R, t), brown (i, r, T), and buff (i, r, t) through the controlled production of different types of anthocyanin and proanthocyanidin pigments. Similar to lentils [85], the main anthocyanin pigments present in black seed-coat soybean is delphinidin-3-monoglucoside [82]. Unfortunately, in lentils, such detailed studies on the pathways/genes governing seed-coat color are lacking. Thus, the identified genotype may prove to be an asset for the identification of the key switches which smoothly regulate the enzymes resulting in such a drastic expression of seed-coat color in the same plant or even in the same branch.

## 4. Materials and Methods

### 4.1. Sowing and Generation-Based Seed-Coat Color Analysis

**Figure 8:**
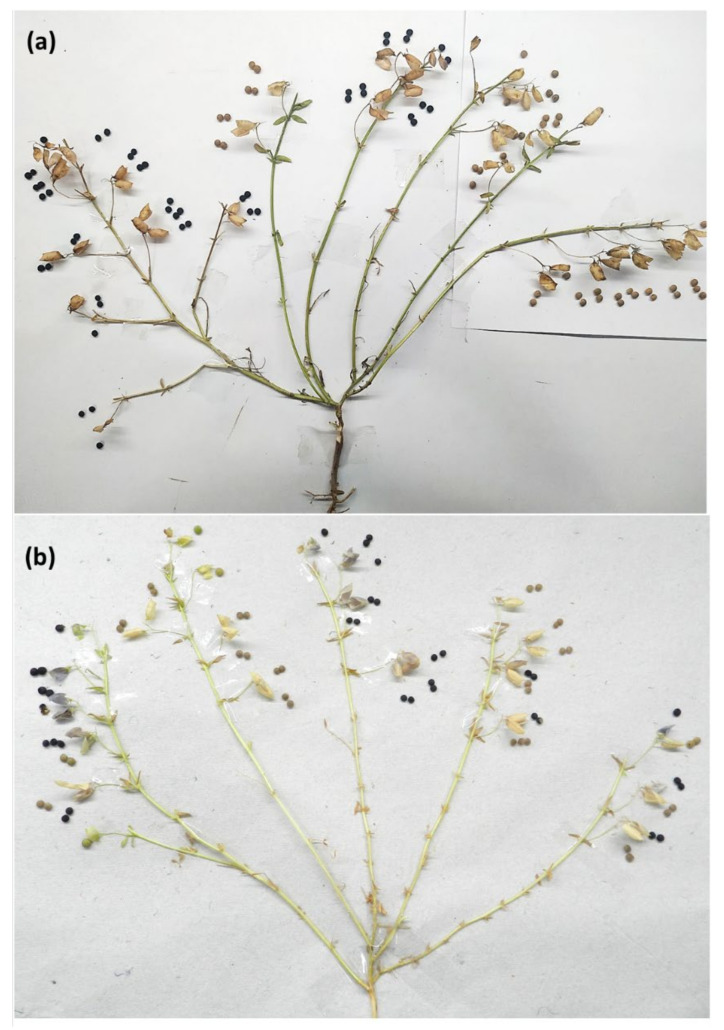
A representative picture of a lentil plant (L4717-NM) showing (**a**) both black and brown seed-coat colored lentil seeds in different branches; (**b**) both black and brown seed-coat colored lentil seeds in the same branch (different pods).

**Figure 9:**
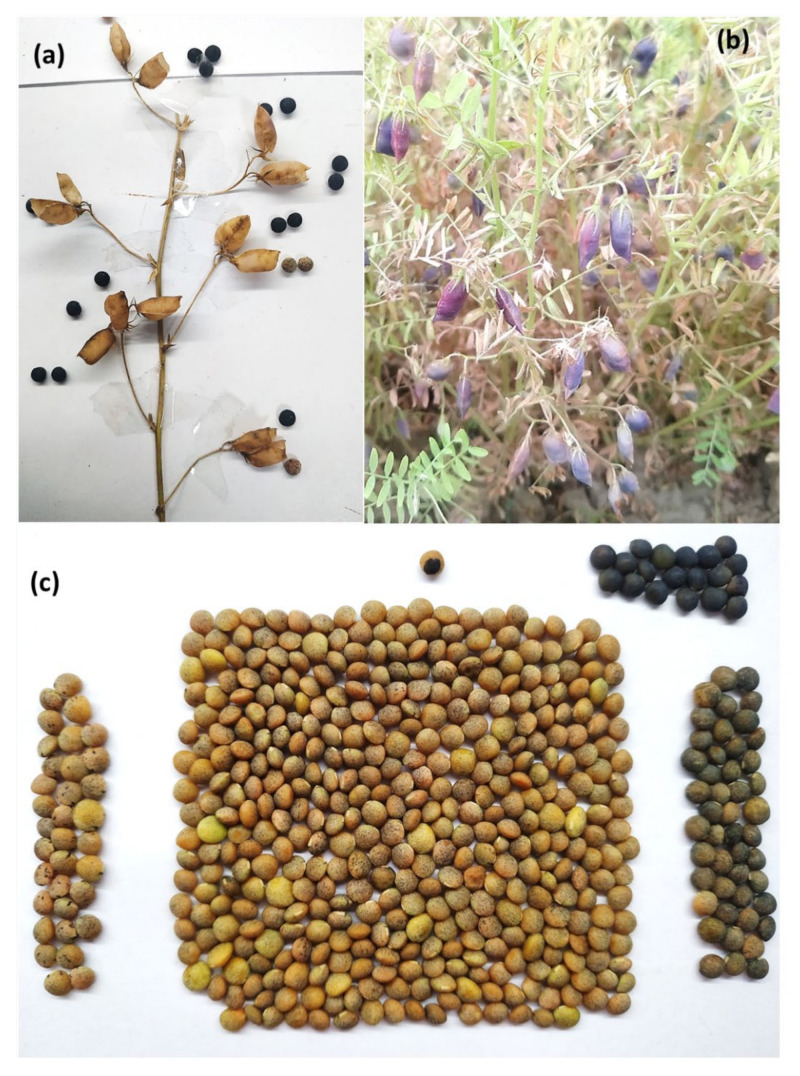
(**a**) A lentil branch showing both black and brown seed-coat color in lentil genotype L4717-NM; (**b**) a lentil plant having black seed-coat lentils with deep purple pigmentation in the pods before full maturity; (**c**) different seed-coat colored lentil seeds produced from a single plant.

A natural mutant plant was identified from an Indian lentil variety named L4717 (Pusa Ageti Masur) in the year 2017–2018 which showed three different types of seeds (namely, brown, black, and spotted) in the same plant (Figure 8 and Figure 9). This unique genotype looks like a natural mutant and was, thus, named L4717-NM (natural mutant). The sowing of lentil genotypes was performed at IARI, New Delhi (28.6331° N and 77.1525° E) during the last week of November each year using standard practice. The row-to row-distance was maintained as 30 cm, while the plant-to-plant spacing was maintained as 5 cm, and the row length was kept as 2 m. Both L4717 and L4717-NM generally mature in 100–103 days under Delhi conditions. Three types of seeds based on the seed-coat color (derived from a single plant of L4717-NM) were again sown during the next year (2018–2019), and data were recorded for the seed-coat color pattern in the individual plants. A similar type of sowing was repeated in the rabi season of the years 2019–2020 and 2020–2021. Data recording was conducted for individual plants. Morphologically, L4717-NM was most similar to the variety L4717.

### 4.2. SSR Marker Analysis

Seedlings of the studied lentil genotypes were grown in a paper towel and subjected to genomic DNA isolation using the CTAB method [90]. The DNA was quantified using NanoDrop 2000c (Thermo Fisher Scientific, Waltham, MA, USA), while a quality check was performed on 0.8% agarose gel, and samples were diluted to 20 ng/μL and then used for PCR based amplification. A total of 54 EST-SSRs [91] which were synthesized from Eurofins Genomics, India (Table S1) were used for the polymorphism survey [92]. The amplified products were separated by electrophoresis (3 h at 100 V in 1 × TBE buffer) on 3% Metaphor agarose gel (Lonza, Rockland, ME, USA). The gel was stained by ethidium bromide and then photographed using a gel documentation system (Alpha Imager) [93].

### 4.3. Biochemical Studies

For antioxidant activity (AoA) studies, we took four different types of seeds, i.e., L4717 along with the brown, black, and spotted seed-coat seeds produced by the identified genotype L4717-NM. The analysis was performed at three different growth stages (seeds, sprouts, and seedlings or microgreens) (Figure 1). Freshly harvested seeds from the year 2019–2020 were used for this analysis. For sprouting, the seeds were wrapped in germination paper, soaked in water, and kept in the dark for 4 days. However, for seedlings, the seeds were again wrapped in germination and butter paper, soaked in water, and kept for 11 days at the National Phytotron Facility (New Delhi) with 21/18 °C as the day/night temperature and natural day and night cycles. The parameters studied were TPC, TFC, DPPH assay, FRAP assay, peroxide quantification, TCC, total crude protein, TAC, and TAA.

#### 4.3.1. Total Phenolic Content (TPC)

A modified Folin–Ciocâlteu colorimetric method [94] was used to determine the TPC. Briefly, 0.5 mL of extract was mixed with 2.5 mL of distilled water, 0.25 mL of 1 N Folin–Ciocâlteu reagent, and 0.75 mL 20% sodium carbonate (Na_2_CO_3_), with the final volume made up to 5 mL. This was then vortexed and incubated at room temperature for 2 h (in dark), and absorbance was measured at 750 nm using a UV/Vis spectrophotometer. Then, a calibration curve of gallic acid (conc. from 10 µg/mL to 60 µg/mL) was generated using a regression equation. The equation of the curve was *y* = 0.0157*x* − 0.0012, and the linearity coefficient was R^2^ = 0.9981. Final values were expressed as mg GAE/100 g DW (dry weight).

#### 4.3.2. Total Flavonoids Content (TFC)

The ethanolic extract was made by crushing 0.05 g of the samples in 80% ethanol (2 mL), and, to this extract (0.5 mL), 2% AlCl_3_ (0.5 mL) was added before incubating for 1 h at room temperature. The absorbance was then measured at 420 nm, and TFC was measured using the calibration curve as quercetin equivalent [95]. The equation of the curve was *y* = 0.0021*x* + 0.004, and the linearity coefficient was R^2^ = 0.9983.

#### 4.3.3. DPPH (1,1-Diphenyl-2-picrylhydrazyl) Scavenging Activity

For the preparation of methanolic extract, 0.05 g of sample was crushed in 2 mL of methanol and centrifuged (12,000 rpm; 10 min). An aliquot (200 µL) was added to 3.8 mL of DPPH and incubated at room temperature for 1 h in dark; absorbance was measured at 517 nm [96]. The DDPH scavenging activity was estimated using the following equation:

(1) $$(\mathrm{DPPH}\;\mathrm{scavenging}\;\mathrm{activity}\;\left(\%\right)=\left[\left({\mathrm{Abs}}_{\mathrm{Control}}\;–{\mathrm{Abs}}_{\mathrm{Sample}}\right)/\left({\mathrm{Abs}}_{\mathrm{Control}}\right)\times 100\right],$$

where, Abs_Control_ is the absorbance of DPPH radical + methanol, and Abs_Sample_ is the absorbance of DPPH radical + sample extract.

#### 4.3.4. FRAP (Ferric Reducing Antioxidant Power) Assay

The sample (0.05 g) was crushed in 80% ethanol (2 mL), the homogenate was centrifuged (12,000 rpm; 15 min), and the supernatant was collected. To 100 µL of extract (per genotype), 3 mL of prewarmed freshly prepared FRAP reagent (10:1:1 of 300 mM acetate buffer (pH 3.6), 10 mM 2,4,6-Tripyridyltriazine (TPTZ), and 20 mM FeCl_3_·6H_2_O) was added. The mixture was incubated (37 °C; 10 min), and an increase in absorbance was measured at 593 nm and compared with the standard calibration curve (20 mM FeSO_4_·7H_2_O). The equation of the curve was *y* = 0.0026*x* + 0.0729, and the linearity coefficient was R^2^ = 0.9657. The final concentration was expressed as µmol/g DW [97].

#### 4.3.5. Peroxide Quantification 

The sample (0.025 g) was homogenized in 1% (*w*/*v*) trichloroacetic acid (1 mL) and centrifuged (12,000 rpm, 15 min, 4 °C). The supernatant (0.75 mL) was then mixed with 10 mM potassium phosphate buffer (0.75 mL; pH 7) and freshly prepared 1 M potassium iodide (1.5 mL) solution. The peroxide quantification was performed by comparing the absorbance at 390 nm with that of the standard calibration curve (10 to 200 µmol/mL of H_2_O_2_), and the concentration was expressed as nmol/g [98].

#### 4.3.6. Total Carotenoid Content (TCC)

The sample (0.05 g) was homogenized in 6 mL of ethyl alcohol/BHT (1 mg of BHT/mL of ethanol), and then incubated (85 °C; 6 min) with continuous vortexing. To this, KOH (120 µL) was added, before incubating (5 min; 85 °C) and cooling on ice. Afterward, distilled water (4 mL) and 3 mL of PE:DE (2:1, *v*/*v*) were added and centrifuged (10 min; 12,000 rpm; room temperature). The upper phase was collected, and distilled water (4 mL) and PE:DE solution (3 mL) were added, before centrifuging. Then, the process was repeated twice, and samples were diluted to 10 mL (PE:DE solution). The increase in absorbance was measured at 470 nm [99]. The TCC was calculated using the following formula:

(2) $$\mathrm{Total}\;\mathrm{carotenoid}\;\mathrm{content}\;\left(\text{µ}g/g\right)=[(A_{\mathrm{total}}\times \;\mathrm{Vol}\;(\mathrm{mL})\;\times \;1000)/(A^{1\%}\;\times \;\mathrm{Sample}\;\mathrm{weight})],$$

where A_total_ = absorbance at 470 nm, Vol (mL) = total volume of extract, and A^1%^ = absorbance coefficient for carotenoid by column mixture.

#### 4.3.7. Total Ascorbic Acid (TAA)

The titration method was used for the ascorbic acid estimation using a 2,6-dichloroindophenol (DCIP) dye solution and 4% oxalic acid as a stabilizing medium [100]. Preparation of the dye solution was performed using NaHCO_3_ hot solution, and dye standardization was performed by titrating the standard ascorbic acid (1 mg/mL) until it showed a pale pink color. This color persisted for 15 s, and final estimation was performed using the following formula:

(3) $$\mathrm{Amount}\;\mathrm{of}\;\mathrm{Ascorbic}\;\mathrm{acid}\;\left(\mathrm{mg}/100\;g\right)=[(X_{\mathrm{mg}}\times \;V_{2}\times \;Z_{\mathrm{mL}})/(V_{1}\times \;Y_{\mathrm{mL}}\times \;\mathrm{Wt}.\;\mathrm{of}\;\mathrm{sample})]\;\times \;100,$$

where X_mg_ = mg of standard ascorbic acid, V_1_ = titer value of standard ascorbic acid against dye, V_2_ = titer value of sample against dye, Y_Ml_ = amount of aliquot taken (mL) for estimation, and Z_mL_ = total amount (mL) of extracted sample.

#### 4.3.8. Total (Crude) Soluble Protein

Total soluble protein was estimated using the Bradford assay [101]. Briefly, 0.05 g of sample was homogenized in 2 mL of phosphate buffer (pH = 7) and centrifuged (13,000 rpm for 20 min); then, to 20 µL of supernatant, 980 µL of distilled water and 2 mL of Bradford reagent were added, before incubating in the dark (10 min) at room temperature. The change in color of Coomassie dye occurs under acidic conditions when protein molecules bind to the dye and the color changes from brown to blue. This color change was then measured at 595 nm by generating a calibration graph using 25, 50, 75, 100, 125, 150, and 200 µg/mL BSA. The equation of the curve was *y* = 0.0029*x* + 0.1418, and the linearity coefficient was R^2^ = 0.9895.

#### 4.3.9. Total Anthocyanin Content (TAC)

Briefly, 0.05 g of sample was crushed in 2 mL of 0.1 N HCl/ethanol 96% (1:9) and centrifuged (6000 rpm, 4 °C, 15 min); the supernatant was diluted with KCl buffer (pH 1) and CH_3_COONa·3H_2_O buffer (pH 4.5) in a 1:4 (sample to buffer) ratio. This was then incubated (15 min in dark), and absorbance was measured at 520 nm and 700 nm [102]. TAC was expressed as delphinidin-3-glucoside equivalent using the following formula:

(4) $$\mathrm{Anthocyanin}\;\mathrm{pigment}\;\left(\mathrm{delphinidin}-3-\mathrm{glucoside}\;\mathrm{equivalents},\;\mathrm{mg}/L\right)=A\;\times \;\mathrm{MW}\;\times \;\mathrm{DF}\;\times \;10^{3}/\varepsilon \;\times \;L\;(1\;\mathrm{cm}),$$

where A = (A_520nm_ − A_700nm_) pH 1 – (A_520nm_ − A_700nm_) pH 4.5, MW (molecular weight) = 500.83 g/mol for delphinidine-3-glucoside (d-3-glu), DF = dilution factor, L = path length in cm, ε = molar extinction coefficient in L mol^–1^·cm^–1^ for (d-3-glu) = 29,000 [103], and 10^3^ = factor for conversion from g to mg.

### 4.4. Statistical Analysis

The experiments were conducted in four replications, and the results were presented as the mean ± SD. SPSS11.5 was used to perform the one-way ANOVA (analysis of variance) to compare the groups, and Pearson’s correlation test was used to assess the correlation between means, while mean comparisons were performed using Tukey’s test. The correlation matrix of metabolites, hierarchical cluster analysis, and heatmap visualization were performed using R software (ver. 4.1.3). PCA (principal component analysis) was performed using the details of various biochemical parameters (TPC, TFC, DPPH, FRAP, H_2_O_2_, TCC, and TAA) in the lentil genotypes differing in terms of seed-coat color. The PCA plot was generated on the basis of the first and second principal components (PC1 and PC2) using STAR ver. 2.0.1.

## 5. Conclusions

**Figure 10:**
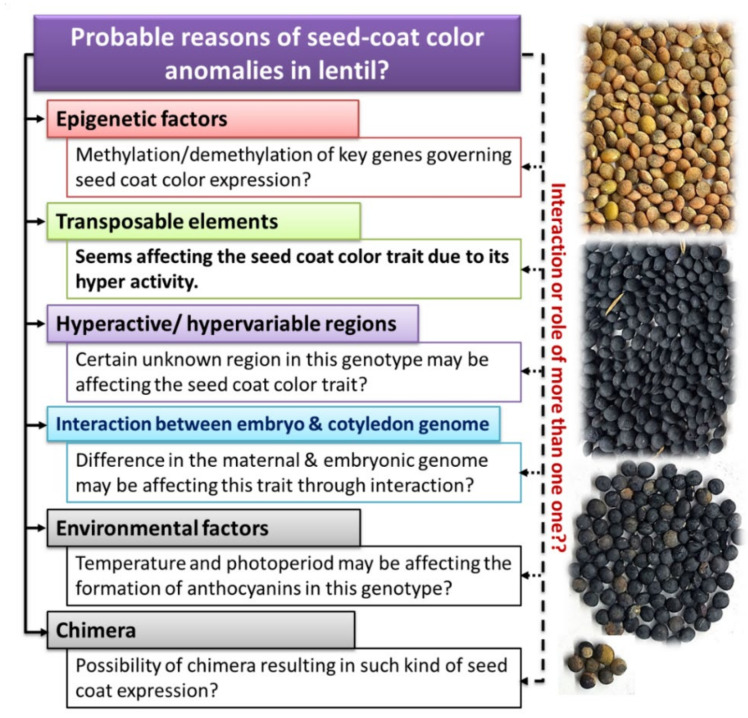
The probable reasons for seed-coat color anomalies in lentil genotype L4717-NM.

The seed coat in the legume crop is derived from the maternal tissue, which is ultimately determined by the genotype of the plant producing the seeds. Therefore, the seed coat in any lentil plant should display only one seed-coat color. On the basis of the results, we strongly presume the possible presence of some hyperactive transposable element regulating seed-coat color in the studied genotype. In addition, the presence of some epigenetic factors regulating the expression of certain key enzymes of the anthocyanin pathway could also be a possibility. Furthermore, there could be some unknown factors or some hypervariable regions which seemingly regulate the anthocyanin pathway in the identified genotype. The presence of environmental factors such as temperature or photoperiod regulating the seed-coat color can be ruled out as even two adjacent pods showed both brown and black seed-coat colored seeds (Figure 10). Interestingly, the seed-coat color and the cotyledon color are unrelated, and the embryo and cotyledon genome may interact and affect the surrounding tissue during morphogenesis in some yet unknown way. Thus, in the future, this genotype may serve as a focal point to understand this unique expression of seed-coat color in lentils. Thus, there is a need to identify a stable black seed-coat genotype which can then be crossed with the fixed brown seed-coat genotype for the formation of a mapping population. Furthermore, a transcriptomic approach can be exploited using black and brown seed-coat genotypes (acting as an isogenic line) at the time of seed formation for the identification of DEGs impacting the trait.

## Figures and Tables

**Table 1:** Overall categorization of plants in different categories (brown, black, and mixed) based on seed-coat color expression.

Year	Seed-Coat Color-Based Plant Category			Total
	Brown Seed-Coat Colored Plants	Black Seed-Coat Colored Plants	Mixed Seed-Coat Colored Plants	
2018–2019	36	01	75	112
2019–2020	75	03	164	242
2020–2021	65	02	102	169

Plants in the year 2018–2019 were the progenies of a single plant which was selected in the year 2017–2018. Similarly, in the years 2019–2020 and 2020–2021, the seeds of a single plant (mixed category) selected from the previous year were used for the generation of progenies.

**Table 2:** Details of seed-coat color expression in 12 plants (2019–2020) each, derived from brown, black, and spotted seed-coat categories (2018–2019) from L4717-NM genotype.

2018–2019 (Brown Seed Coat)	2019–2020		2018–2019 (Black Seed Coat)	2019–2020		2018–2019 (Spotted Seed Coat)	2019–2020	
	Progenies	Seed-Coat Color		Progenies	Seed-Coat Color		Progenies	Seed-Coat Color
Plant-1	SP-1	Brown	Plant-1	SP-1	Mixed	Plant-1	SP-1	Brown
	SP-2	Brown		SP-2	Brown		SP-2	Mixed
	SP-3	Mixed		SP-3	Mixed		SP-3	Brown
Plant-2	SP-1	Black	Plant-2	SP-1	Brown	Plant-2	SP-1	Mixed
	SP-2	Mixed		SP-2	Mixed		SP-2	Mixed
	SP-3	Mixed		SP-3	Brown		SP-3	Mixed
Plant-3	SP-1	Mixed	Plant-3	SP-1	Mixed	Plant-3	SP-1	Mixed
	SP-2	Mixed		SP-2	Mixed		SP-2	Mixed
	SP-3	Mixed		SP-3	Mixed		SP-3	Mixed
Plant-4	SP-1	Mixed	Plant-4	SP-1	Mixed	Plant-4	SP-1	Brown
	SP-2	Mixed		SP-2	Mixed		SP-2	Mixed
	SP-3	Mixed		SP-3	Mixed		SP-3	Mixed

SP: single plant; spotted seeds were kept in the black category. Since there was only one black and no pure spotted seed-coat plants identified, we took the black and spotted seeds from the mixed category of plants. The photographs of the seeds derived from these single plants are presented in Figure 2, Figure 3 and Figure 4.

**Table 3:** Biochemical characterization of lentil genotypes differing for the seed coat color.

S. No.	Samples	TPC (mg GAE/100 g DW)	TFC (mg/g DW)	DPPH scavenging (%)	FRAP (µmol Fe^2+^/g DW)	H_2_O_2_ (nmol/g DW)	TCC (mg/100 g DW)	Crude protein (g/100 g DW)	TAC (mg/g DW)	TAA (mg/100 g DW)
1	L4717 seeds	1108.15 ± 32.12 h	**1.91 ± 0.07 d**	**35.97 ± 4.67 d**	8.56 ± 0.66 e	**1.64 ± 0.05 c**	**2.55 ± 0.09 e**	25.60 ± 0.05 ab	**0.016 ± 0.009 b**	27.94 ± 1.70 fg
2	Brown seed- coat seeds	1206.24 ± 20.53 g	1.97 ± 0.02 cd	42.86 ± 3.63 cd	9.23 ± 0.64 e	1.76 ± 0.08 c	2.57 ± 0.10 e	25.04 ± 0.36 b	0.018 ± 0.003 b	29.41 ± 4.80 defg
3	Black seed- coat seeds	1132.99 ± 44.25 h	2.04 ± 0.17 cd	36.48 ± 3.67 d	**7.35 ± 0.20 f**	1.78 ± 0.14 c	2.61 ± 0.11 e	24.88 ± 0.29 b	**0.042 ± 0.018 a**	28.68 ± 5.02 efg
4	Spotted seed- coat seeds	**1047.64 ± 32.29 i**	1.98 ± 0.29 cd	44.90 ± 5.46 c	8.82 ± 0.60 e	1.79 ± 005 c	2.57 ± 0.14 e	**26.43 ± 1.13 a**	0.033 ± 0.004 ab	**27.21 ± 2.82 g**
5	L4717 sprouts	1489.68 ± 11.86 f	2.41 ± 0.12 bc	59.45 ± 2.31 ab	14.37 ± 0.09 d	2.84 ± 0.14 a	41.95 ± 0.30 bc	21.75 ± 0.11 d	ND	35.29 ± 0.00 cdef
6	Brown seed- coat sprouts	1471.21 ± 12.72 f	2.62 ± 0.13 b	46.94 ± 3.23 c	14.99 ± 0.10 c	2.95 ± 0.23 a	42.81 ± 0.21 b	21.16 ± 0.45 d	ND	38.24 ± 2.40 abc
7	Black seed- coat sprouts	1639.36 ± 17.94 e	2.53 ± 0.27 b	41.92 ± 2.36 cd	14.75 ± 0.17 d	2.93 ± 0.02 a	**45.35 ± 1.44 a**	20.80 ± 0.31 d	ND	36.03 ± 1.47 bcde
8	Spotted seed- coat sprouts	1628.54 ± 18.95 e	2.39 ± 0.32 bc	47.10 ± 3.12 c	14.75 ± 0.40 d	**3.04 ± 0.16 a**	42.72 ± 0.29 b	23.62 ± 0.54 c	ND	36.76 ± 2.94 a bcd
9	L4717 seedlings	2107.52 ± 21.72 d	3.25 ± 0.26 a	**66.46 ± 1.00 a**	**46.04 ± 0.34 a**	2.74 ± 0.17 a	37.95 ± 0.30 bd	9.82 ± 0.16 f	ND	42.65 ± 3.80 abc
10	Brown seed- coat seedlings	2332.36 ± 25.76 b	3.09 ± 0.25 a	56.55 ± 2.83 b	44.70 ± 1.59 b	2.81 ± 0.12 a	38.81 ± 0.21 d	**9.52 ± 013 f**	ND	**44.12 ± 3.40 a**
11	Black seed- coat seedlings	**2434.27 ± 14.62 a**	3.15 ± 0.17 a	61.89 ± 3.68 ab	43.24 ± 0.68 c	2.41 ± 0.07 b	41.35 ± 1.44 c	10.80 ± 0.15 e	ND	43.97 ± 2.42 a
12	Spotted seed- coat seedlings	2199.24 ± 16.25 c	**3.28 ± 0.07 a**	65.85 ± 1.22 a	42.85 ± 0.36 c	2.30 ± 0.07 b	38.72 ± 0.29 d	9.93 ± 0.57 ef	ND	43.38 ± 4.52 ab

TPC: total phenolic content, TFC: total flavonoid content, DPPH: 2,2-diphenyl-1-picrylhydrazyl, FRAP: ferric reducing antioxidant power, H_2_O_2_: peroxide quantification, TCC: total carotenoid content, TAC: total anthocyanin content, TAA: total ascorbic acid. Values are expressed as mean ± SD (n = 3) and different letters indicate a significant difference (*p* ≤ 0.05). Bold letters represent the maximum and minimum values and ND: not detected.

## Data Availability

Data are contained within the article or Supplementary Materials.

## Supplementary Materials

The following supporting information can be downloaded at https://www.mdpi.com/article/10.3390/plants11141815/s1: Figure S1. A representative picture showing the amplification details in four lentil samples (differing in terms of seed-coat color) using SSR markers. PLC2, 12, 10, and 11 are the primers; 1: L4717, 2: brown seed coat, 3: black seed coat, 4: spotted seed coat (2–4 were obtained from the L4717-NM expressing a mixed phenotype); Table S1: List of primers used in the study (Jain et al. 2013).

## References

[1] Mishra G.P., Aski M.S., Bosamia T., Chaurasia S., Mishra D.C., Bhati J., Kumar A., Javeria S., Tripathi K., Kohli M. (2022). Insights into the host-pathogen interaction pathways through RNA-Seq analysis of *Lens culinaris* Medik. in response to *Rhizoctonia bataticola* infection. Genes.

[2] (2021). FAOSTAT. https://www.fao.org/faostat/en/#home.

[3] Mishra G.P., Dikshit H.K., Kumari J., Priti, Tripathi K., Devi J., Aski M., Mehra R., Sarker A., Kumar S. (2020). Identification and characterization of novel Penta-podded genotypes in the cultivated lentil (*Lens culinaris* Medik.). Crop Sci..

[4] Tschermak-Seysenegg E. (1928). Lentil and Field bean crosses. Sityringsber Akad. Wiss. Wein Math. Nat. KI.I. Abt..

[5] Ladizinsky G. (1979). The genetics of several morphological traits in the lentil. J. Hered..

[6] Erskine W., Witcomb J.R. (1984). Lentil Germplasm Catalogue.

[7] Emami M.K., Sharma B. (2000). Inheritance of black testa color in lentil (*Lens culinaris* Medik). Euphytica.

[8] Wilson V.E., Hudson L.W. (1978). Inheritance of lentil flower color. J. Hered..

[9] Vandenberg A., Slinkard A.E. (1990). Genetics of seed coat colour and pattern in lentil. J. Hered..

[10] Slinkard A.E. (1978). Inheritance of cotyledon color in lentils. J. Hered..

[11] Sharma B., Emami M.K. (2002). Discovery of a new gene causing dark green cotyledon and pathway of pigment synthesis in lentil (*Lens culinaris* Medil). Euphytica.

[12] Subedi M., Tabil L.G. (2018). Vandenberg A Influence of seed coat color genes on milling qualities of red lentil (*Lens culinaris* Medik). J. Agril. Sci..

[13] Mirali M., Purves R.W., Stonehouse R., Song R., Bett K., Vandenberg A. (2016). Genetics and biochemistry of zero-tannin lentils. PLoS ONE.

[14] Vaillancourt R., Slinkard A.E., Reichert R.D. (1986). The inheritance of condensed tannin concentration in lentil. Can. J. Plant Sci..

[15] Miyamoto T., Everson E.H. (1958). Biochemical and physiological studies of wheat seed pigmentation. Agron. J..

[16] Styles E.D., Ceska O. (1977). The genetic control of flavonoid synthesis in maize. Can J. Genet. Cytol..

[17] McCallum J.A., Walker J.R.L. (1990). Proanthocyanidins in Wheat Bran. Cereal Chem..

[18] Finocchiaro F., Ferrari B., Gianinetti A. (2010). A study of biodiversity of flavonoid content in the rice caryopsis evidencing simultaneous accumulation of anthocyanins and proanthocyanidins in a black-grained genotype. J. Cereal Sci..

[19] Kohyama N., Chono M., Nakagawa H., Matsuo Y., Ono H., Matsunaka H. (2017). Flavonoid compounds related to seed coat color of wheat. Biosci. Biotechnol. Biochem..

[20] Mannino G., Gentile C., Ertani A., Serio G., Bertea C.M. (2021). Anthocyanins: Biosynthesis, Distribution, Ecological Role, and Use of Biostimulants to Increase Their Content in Plant Foods—A Review. Agriculture.

[21] Yue E., Huang Y., Qian L., Lu Q., Wang X., Qian H., Yan J., Ruan S. (2022). Comparative Analysis of Proanthocyanidin Metabolism and Genes Regulatory Network in Fresh Leaves of Two Different Ecotypes of *Tetrastigma hemsleyanum*. Plants.

[22] Holton T.A., Cornish E.C. (1995). Genetics and Biochemistry of Anthocyanin Biosynthesis. Plant Cell.

[23] Mannino G., Chinigò G., Serio G., Genova T., Gentile C., Munaron L., Bertea C.M. (2021). Proanthocyanidins and Where to Find Them: A Meta-Analytic Approach to Investigate Their Chemistry, Biosynthesis, Distribution, and Effect on Human Health. Antioxidants.

[24] Qin Z., Liu H.-M., Ma Y.-X., Wang X.-D. (2021). Developments in extraction, purification, and structural elucidation of proanthocyanidins (2000–2019). Stud. Nat. Prod. Chem..

[25] Zhu F. (2019). Proanthocyanidins in cereals and pseudocereals. Crit. Rev. Food Sci. Nutr..

[26] Jende-Strid B. (1993). Genetic control of flavonoid biosynthesis in barley. Hereditas.

[27] Trojan V., Musilova M., Vyhnanek T., Klejdus B., Hanadek P., Havel L. (2014). Chalcone synthase expression and pigment deposition in wheat with purple and blue colored caryopsis. J. Cereal. Sci..

[28] Shoeva O.Y., Mock H.P., Kukoeva T.V., Börner A., Khlestkina E.K. (2016). Regulation of the Flavonoid Biosynthesis Pathway Genes in Purple and Black Grains of *Hordeum vulgare*. PLoS ONE.

[29] Nozzolillo C., Ricciardi L., Hemingway R.W., Laks P.E. (1992). Proanthocyanidin Content of Broad Bean Seeds: Relationship of Seed Coat Color. Plant Polyphenols, Basic Life Sciences.

[30] Todd J.J., Vodkin L.O. (1993). Pigmented Soybean (*Glycine max*) Seed Coats Accumulate Proanthocyanidins during Development. Plant Physiol..

[31] Mirali M., Purves R.W., Vandenberg A. (2017). Profiling the phenolic compounds of the four major seed coat types and their relation to color genes in lentil. J. Nat. Prod..

[32] Wilson V.E., Hudson L.W. (1978). Seedcoat color anomalies in early generations of lentils. J. Hered..

[33] Wilson V.E., Hudson L.W. (1980). Lentil seedcoat background color inheritance. J. Hered..

[34] Narendra V.G., Abdorrazzaghi M. (2013). An Intelligent system for identification of Indian Lentil types using Artificial Neural Network (BPNN). IOSR J. Comp. Engg..

[35] Duenas M., Sun B., Hernaändez T., Estrella I., Spranger M.I. (2003). Proanthocyanidin composition in the seed coat of lentils (*Lens*
*culinaris* L.). J. Agric. Food Chem..

[36] Bakhsh A., Iqbal S.M., Cheema N.M. (2013). Inheritance of morphological characters associated with plant and dried seeds in lentil (*Lens culinaris* Medik.). Pak. J. Bot..

[37] Prakken R. (1970). Inheritance of colour in *Phaseolus vulgaris* L. II. A critical review. Meded. Landbouwhogesch Wagening..

[38] Harland S.C. (1919). Inheritance of certain characters in the cowpea (*Vigna sinensis*). J. Genet..

[39] Fruwirth C. (1917). Selection in pure lines. J. Hered..

[40] Imai Y. (1935). Recurrent Pseudomutation. Am. Nat..

[41] Jambhale N.D., Nerkar Y.S. (1985). An unstable gene controlling developmental variegation in okra (*Abelmoschus esculentus* (L.) Moench). Theoret. Appl. Genet..

[42] Punia S.S., Ram B., Dheer M., Jain N.K., Koli N.R., Khedar O.P. (2014). Hyper-variable spontaneous genetic variation for earliness, seed characters and other yield-contributing traits in lentil (*Lens culinaris* Med.). Curr. Sci..

[43] Wilson V.E. (1972). Morphology and technique for crossing *Lens esculenta* Moench. Crop Sci..

[44] Kumar A., Singh D.P. (1998). Hybridization technique in lentil under field conditions. Lens Newslett..

[45] Wilson V.E., Law A.G. (1972). Natural crossing in *Lens esculenta* Moench. J. Am. Soc. Hortic. Sci..

[46] Chandlee J.M., Vodkin L.O. (1989). Unstable expression of a soybean gene during seed coat development. Theoret. Appl. Genet..

[47] Groose R.W., Schulte S.M., Palmer R.G. (1990). Germinal reversion of an unstable mutation for anthocyanin pigmentation in soybean. Theoret. Appl. Genet..

[48] Zabala G., Vodkin L.O. (2014). Methylation Affects Transposition and Splicing of a Large CACTA Transposon from a MYB Transcription Factor Regulating Anthocyanin Synthase Genes in Soybean Seed Coats. PLoS ONE.

[49] Prior R.L., Wu X., Schaich K. (2005). Standardized methods for the determination of antioxidant capacity and phenolics in foods and dietary supplements. J. Agric. Food Chem..

[50] Heim K.E., Tagliaferro A.R., Bobilya D.J. (2002). Flavonoid antioxidants: Chemistry, metabolism and structure-activity relationships. J. Nutr. Biochem..

[51] Aron P.M., Kennedy J.A. (2008). Flavan-3-ols: Nature, occurrence and biological activity. Mol. Nutr. Food Res..

[52] Swieca M., Surdyka M., Gawlik-Dziki U., Złotek U., Baraniak B. (2014). Antioxidant potential of fresh and stored lentil sprouts affected by elicitation with temperature stresses. Int. J. Food Sci. Technol..

[53] Fouad A.A., Rehab F.M.A. (2015). Effect of germination time on proximate analysis, bioactive compounds and antioxidant activity of lentil (*Lens culinaris* Medik.) sprouts. Acta Sci. Pol. Technol. Aliment..

[54] Altuner F. (2021). Determination of biochemical composition and pigment content in legume and cereal microgreens. Legume Res..

[55] Fuente B.D.L., López-García G., Máñez V., Alegría A., Barberá R., Cilla A. (2019). Evaluation of the bioaccessibility of antioxidant bioactive compounds and minerals of four genotypes of brassicaceae microgreens. Foods.

[56] Priti, Mishra G.P., Dikshit H.K., Vinutha T., Tontang M.T., Stobdan T., Sangwan S., Aski M., Singh A., Kumar R.R. (2021). Diversity in phytochemical composition, antioxidant capacities, and nutrient contents among mungbean and lentil microgreens when grown at plain-altitude region (Delhi) and high-altitude region (Leh-Ladakh), India. Front. Plant Sci..

[57] Swieca M., Gwalik-Dziki U. (2015). Effects of sprouting and postharvest storage under cool temperature conditions on starch content and antioxidant capacity of green pea, lentil and young mung bean sprouts. Food Chem..

[58] Ahuja H., Kaur S., Gupta A.K., Singh S., Kaur J. (2015). Biochemical mapping of lentil (*Lens culinaris* Medik) genotypes for quality traits. Acta Physiol. Plant..

[59] Wojdylo A., Nowicka P., Tkacz K., Turkiewicz I.P. (2020). Sprouts vs. microgreens as novel functional foods: Variation of nutritional and phytochemical profiles and their in vitro bioactive properties. Molecules.

[60] Cheeseman J.M. (2006). Hydrogen peroxide concentrations in leaves under natural conditions. J. Exp. Bot..

[61] Ozden M., Demirel U., Kahraman A. (2009). Effects of proline on antioxidant system in leaves of grapevine (*Vitis vinifera* L.) exposed to oxidative stress by H_2_O_2_. Sci. Hortic..

[62] Biju S., Fuentes S., Gupta G. (2018). Physiological and Biochemical Responses of Lentils to Silicon Mediated Drought Tolerance. https://grdc.com.au/resources-and-publications/grdc-update-papers/tab-content/grdc-update-papers/2018/02/physiological-and-biochemical-responses-of-lentils-to-silicon-mediated-drought-tolerance.

[63] Yang C., Shahidi F., Tsao R., Apak R., Capanoglu E., Shahidi F., Hoboken N.J. (2018). Biomarkers of oxidative stress and cellular-based assays of indirect antioxidant measurement. Measurement of Antioxidant Activity and Capacity: Recent Trends and Applications.

[64] Zhang B., Deng Z., Tang Y., Chen P., Liu R., Ramdath D.D., Liu Q., Hernandez M., Tsao R. (2014). Fatty acid, carotenoid and tocopherol compositions of 20 Canadian lentil cultivars and synergistic contribution to antioxidant activities. Food Chem..

[65] Ashokkumar K., Diapari M., Jha A.B., Tar’an B., Arganosa G., Warkentin T.D. (2015). Genetic diversity of nutritionally important carotenoids in 94 pea and 121 chickpea accessions. J. Food Compos. Anal..

[66] Kowitcharoen L., Phornvillay S., Lekkham P., Pongprasert N., Srilaong V. (2021). Bioactive composition and nutritional profile of microgreens cultivated in Thailand. Appl. Sci..

[67] Swieca M., Baraniak B. (2014). Nutritional and Antioxidant Potential of Lentil Sprouts Affected by Elicitation with Temperature Stress. J. Agric. Food Chem..

[68] Smirnoff N., Wheeler G.L. (2000). Ascorbic acid in plants: Biosynthesis and function. Critical. Rev. Plant Sci..

[69] Paciolla C., Fortunato S., Dipierro N., Paradiso A., Leonardis S.D., Mastropasqua L. (2019). Pinto MCD Vitamin C in plants: From functions to biofortification. Antioxidants.

[70] Fidrianny I., Suhendy H., Insanu M. (2018). Correlation of phytochemical content with antioxidant potential of various sweet potato (*Ipomoea batatas*) in West Java, Indonesia. Asian Pac. J. Trop. Biomed..

[71] Bhoyar M.S., Mishra G.P., Naik P.K., Srivastava R.B. (2011). Estimation of antioxidant activity and total phenolics among natural populations of Caper (*Capparis spinosa*) leaves collected from cold arid desert of trans-Himalayas. Aust. J. Crop Sci..

[72] Bhoyar M.S., Mishra G.P., Naik P.K., Singh S.B. (2018). Evaluation of antioxidant capacities and total polyphenols in various edible parts of *Capparis spinosa* L. collected from trans-Himalayas. Def. Life Sci. J..

[73] Patel K.G., Mandaliya V.B., Mishra G.P., Dobaria J.R., Radhakrishnan T. (2016). Transgenic peanut overexpressing *mtlD* gene confers enhanced salinitystress tolerance via mannitol accumulation and differential antioxidative responses. Acta Physiol. Plant.

[74] Bhalani H., Mishra G.P., Sarkar T., Radhakrishnan T. (2019). Regulation of antioxidant mechanisms by AtDREB1A improves soil-moisture deficit stress tolerance in transgenic peanut (*Arachis hypogaea* L.). PLoS ONE.

[75] Devi J., Sanwal S.K., Koley T.K., Mishra G.P., Karmakar P., Singh P., Singh B. (2019). Variations in the total phenolics and antioxidant activities among garden pea (*Pisum sativum* L.) genotypes differing for maturity duration, seed and flower traits and their association with the yield. Sci. Hortic..

[76] Pereira D.M., Valentao P., Pereira J.A., Andrade P.B. (2009). Phenolics: From chemistry to biology. Molecules.

[77] Oktay M., Gulcin I., Kufrevioglu O.I. (2003). Determination of in vitro antioxidant activity of fennel (*Foeniculum*
*vulgare*) seed extracts. LebensmWiss Techol..

[78] El-Nakhel C., Pannico A., Kyriacou M.C., Giordano M., De Pascale S., Rouphael Y. (2019). Macronutrient deprivation eustress elicits differential secondary metabolites in red and green-pigmented butterhead lettuce grown in a closed soilless system. J. Sci. Food Agric..

[79] El-Nakhel C., Giordano M., Pannico A., Carillo P., Fusco G.M., De Pascale S., Rouphael Y. (2019). Cultivar-specific performance and qualitative descriptors for butterhead Salanova lettuce produced in closed soilless cultivation as a candidate salad crop for human life support in space. Life.

[80] Kyriacou M.C., El-Nakhel C., Pannico A., Graziani G., Soteriou G.A., Giordano M., Zarrelli A., Ritieni A., De Pascale S., Rouphael Y. (2019). Genotype-specific modulatory effects of select spectral bandwidths on the nutritive and phytochemical composition of microgreens. Front. Plant Sci..

[81] El-Nakhel C., Pannico A., Graziani G., Kyriacou M.C., Giordano M., Ritieni A., De Pascale S., Rouphael Y. (2020). Variation in macronutrient content, phytochemical constitution and in vitro antioxidant capacity of green and red butterhead lettuce dictated by different developmental stages of harvest maturity. Antioxidants.

[82] Yang K., Jeong N., Moon J.K., Lee Y.H., Lee S.H., Kim H.M., Hwang C.H., Back K., Palmer R.G., Jeong S.C. (2010). Genetic analysis of genes controlling natural variation of seed coat and flower colors in soybean. J. Hered..

[83] Khoo H.E., Azlan A., Tang S.T., Lim S.M. (2017). Anthocyanidins and anthocyanins: Colored pigments as food, pharmaceutical ingredients, and the potential health benefits. Food Nutr. Res..

[84] Quattrocchio F., Baudry A., Lepiniec L., Grotewold E., Grotewold E. (2016). The regulation of flavonoid biosynthesis. The Science of Flavonoids.

[85] Takeoka G.R., Dao L.T., Tamura H., Harden L.A. (2005). Delphinidin 3-O-(2-O-â-D-Glucopyranosyl-r-L-arabinopyranoside): A novel anthocyanin identified in beluga black lentils. J. Agric. Food Chem..

[86] Oomah B.D., Caspar F., Malcolmson L.J., Bellido A.-S. (2011). Phenolics and antioxidant activity of lentil and pea hulls. Food Res. Int..

[87] Xu W., Grain D., Bobet S., Le Gourrierec J., Thévenin J., Kelemen Z., Lepiniec L., Dubos C. (2014). Complexity and robustness of the flavonoid transcriptional regulatory network revealed by comprehensive analyses of MYB-bHLH-WDR complexes and their targets in Arabidopsis seed. New Phytol..

[88] Ichino T., Fuji K., Ueda H., Takahashi H., Koumoto Y., Takagi J., Tamura K., Sasaki R., Aoki K., Shimada T. (2014). GFS9/TT9 contributes to intracellular membrane trafficking and flavonoid accumulation in *Arabidopsis thaliana*. Plant J..

[89] Ren Y., He Q., Ma X., Zhang L. (2017). Characteristics of Color Development in Seeds of Brownand Yellow-Seeded Heading Chinese Cabbage and Molecular Analysis of Brsc, the Candidate Gene Controlling Seed Coat Color. Front. Plant Sci..

[90] Murray M., Thompson W. (1980). The isolation of high molecular weight plant DNA. Nucleic Acids Res..

[91] Jain N., Dikshit H.K., Singh D., Singh A., Kumar H. (2013). Discovery of EST-derived microsatellite primers in the legume *Lens culinaris* (Fabaceae). Appl. Plant Sci..

[92] Dasgupta U., Mishra G.P., Dikshit H.K., Mishra D.C., Bosamia T., Roy A., Bhati J., Priti, Aski M., Kumar R.R. (2021). Comparative RNA-Seq analysis unfolds a complex regulatory network imparting yellow mosaic disease resistance in mungbean [*Vigna radiata* (L.) R. Wilczek]. PLoS ONE.

[93] Kumar H., Singh A., Dikshit H.K., Mishra G.P., Aski M., Meena M.C., Kumar S. (2019). Genetic dissection of grain iron and zinc concentrations in lentil (*Lens culinaris* Medik.). J. Genet..

[94] Singleton V.L., Orthofer R., Lamuela-Raventos R.M. (1999). Analysis of total phenols and other oxidation substrates and antioxidants by means of Folin-Ciocalteu reagent. Methods Enzymol..

[95] Woisky R.G., Salatino A. (1998). Analysis of propolis: Some parameters and procedure for chemical quality control. J. Apicultural. Res..

[96] Brand-Williams W., Cuvelier M.E., Berset C. (1995). Use of a free radical method. LWT-Food Sci. Technol..

[97] Benzie F.F., Strain J.J. (1999). Ferric Reducing/antioxidant power assay: Direct measure of total antioxidant activity of biological fluids and modified version for simultaneous measurement of total antioxidant power and ascorbic acid concentration. Methods Enzymol..

[98] Loreto F., Velikova V. (2001). Isoprene produced by leaves protects the photosynthetic apparatus against ozone damage, quenches ozone products, and reduces lipid peroxidation of cellular membranes. Plant Physiol..

[99] Lichtenthaler H.K., Wellburn A.R. (1983). Determination of total carotenoids and chlorophylls A and B of leaf in different solvents. Biol. Soc. Trans..

[100] Nielsen S.S., Vitamin C. (2017). Determination by Indophenol method. Food Analysis Laboratory Manual.

[101] Bradford M.M. (1976). A rapid and sensitive method for the quantitation of microgram quantities of protein utilizing the principle of protein dye binding. Anal Biochem..

[102] Lee J., Durst R.W., Wrolstad R.E. (2005). Determination of total monomeric anthocyanin pigment content of fruit juices, beverages, natural colorants, and wines by the pH differential method: Collaborative Study. J AOAC Int..

[103] Giusti M.M., Luis E., Saona R., Wrolstad R.E. (1999). Molar absorptivity and color characteristics of acylated and non-acylated pelargonidin-based anthocyanins. J. Agric. Food Chem..

