# Supplementary material for: Morphological, Molecular, and Biochemical Characterization of a Unique Lentil (Lens culinaris Medik.) Genotype Showing Seed-Coat Color Anomalies Due to Altered Anthocyanin Pathway
Source: Plants (Basel). 2022 Jul 10;11(14):1815. doi: 10.3390/plants11141815 (PMC9319573; doi:10.3390/plants11141815)
Supplement: Supplementary file 1 [file plants-11-01815-s001.zip › plants-1713624-supplementary.pdf]

Table S1. List of primers used in the study (Jain *et al.* 2013)

S. No.	Primer	Sequence (5'-3')
1.	PLC 1	F: TCA TTC CTT CAT CTT CCA CGT A<br>R: TTT GAC TTT CCA ACA ACC TCC T
2.	PLC 2	F: TTG ACT GTT CTG GCG TTT TCT A<br>R: TGC ACC ATC TTT TGC CTA CAT A
3.	PLC 3	F: AGA TCG TTT GCT CAT CTG GAA T<br>R: TCC TTT TCC TTC ATT AAC GGT G
4.	PLC 4	F: CCTATCGGGAAACTACATGGAA<br>R: TCT GCA TTG GTC TTC TTC TCA A
5.	PLC 7	F: GCT TTT ATG ATC TTC TCG TGG T<br>R: CGA GGA TTA CTT TTC AAT GGT C
6.	PLC 8	F: CTC CTT CCA TTT CTC TTT CTG C<br>R: TCC TGA ACG ACA CCA ACA CTA C
7.	PLC 10	F: TGC AAC AAA GGA CAC TAG AGG TT<br>R: ATT TCT TTC TCC CTA ACC AGC C
8.	PLC 12	F: GGA AGC AAG ATG GAA GAA GTT G<br>R: GCG CCA TTA GTG CAG AGT AAA T
9.	PLC 14	F: TCT GGA AGA GGG TTT GTA CCA T<br>R: GCA GTT AGA TCA CAG CTA CCA AAA
10.	PLC 16	F: CGT TTG ATC TTC TAA GCC CCT A<br>R: AAG GGA AAG GAT GTT TGA CTT G
11.	PLC 18	F: GGA CCA TCA ACT AGC ACA TGA C<br>R: TCA CAT CAT CAA CAT GCT CAA C
12.	PLC 20	F: GGT CTT TTA TGG GAA GTG CTT G<br>R: TTA ACA AAC AGG CTA GGC CAA T
13.	PLC 22	F: TAC ACT GAA GGA GAT GCA CTG G<br>R: TAA CAA CAA AAC ACA GCT TCG C
14.	PLC 24	F: CAT GTT AAT GAC GGG TAG CTG A<br>R: TGA TTT TGC CTT GTG TGG TAT C
15.	PLC 27	F: AAA TAG TGT TGC TGG ATT GGC T<br>R: ACC TTT CCT ATC TTG TTT CCC A
16.	PLC 29	F: TAC TTT CCA CAA AAC TCG CAG A<br>R: CAA CAA CAT GCA AAC AAC ACA G
17.	PLC 30	F: TTG GTC AGG TTC TCA ATC CTC T<br>R: ACG GAT GAA CGC TTG TAA AGA A
18.	PLC 31	F: CAT TGC AGC TTA TTC TCA CAG C<br>R: TGA CCC ATC CTC ATC CTT AAA T
19.	PLC 33	F: CTA AAG ATG AGC ATT TGG ACC C<br>R: TGT TCA CCA CCA AGA ATG GTA A
20.	PLC 34	F: TAC TGG ATG AGA CGA AGA TGG A<br>R: CGA AAC CTG GCC TAT ACA AAA G
21.	PLC 35	F: TTG CTT CCT CCT CTT CTC ACT C<br>R: AGC CTC AGT ACC CTC CTC TTT T
22.	PLC 36	F: ACT CAA GTC AAC CTC AGA AGG C<br>R: CTT AGG AGC CGG AGA AGAAGA T
23.	PLC 37	F: CTC TCC AGT CCT TGC TTG ATG<br>R: ACC AAC AAA CTT GCC AGA CTT C
24.	PLC 38	F: CCT GGA GAA GTC TGT GGA AGA T<br>R: AGC TCT AGC ATT TTG CAT GTG A
25.	PLC 39	F: CAG AGA AAT CCC CTG CTG AG<br>R: CAT GAT TCC CAT AGC CTT GC
26.	PLC 40	F: CAA CTC GCA TCC TCT TCA CA<br>R: CAA AGG GGT TGG AGT CGT AA
27.	PLC 43	F: CGT GTG GTC CTA TCC TCT TG<br>R: ACG ATC AAA AGA AAA CCC GC
28.	PLC 45	F: CCT TAG TCA CTG TGG TCG ATG A

		R: ACA ATG AGA GGC CAG TGC TT
29.	PLC 46	F: CAA ACT GGA AGA TGC TGC TG<br>R: TGA CCC ATC CTC ATC CTT AAA
30.	PLC 48	F: TGT GGT ACA TGC ACA CCA AAT<br>R: GGT GGT AGC AGT GGT GGA GT
31.	PLC 49	F: TTG TTT TGA GAA CCT TCC CC<br>R: TTT TGC AAG GGT ATT TCT TTT TG
32.	PLC 50	F: CGA TTG GTC TTA TAT GGT TCT G<br>R: AAG CTA CCT GCA TAC TTG GTC
33.	PLC 51	F: CCA TGA TGA GCC TTG AAT GA<br>R: TCT TCA ATC TCC AGG AAC ACT TT
34.	PLC 53	F: TCG TGA TAA AAA CGG GGA AG<br>R: TAT CTT TGC CAC TGC CTC CT
35.	PLC 54	F: GTA AAC GAA GCT CAG AGC CG<br>R: CAT ATC CAC GAT CCC TGC TT
36.	PLC 55	F: AGA CAC CGG CAT CAA ATC AT<br>R: CAT ATT CAA ATA TTC AGT GTC ATG TTC
37.	PLC 56	F: GCC ATT TGT TGTTGT GTT TCA<br>R: TGC CAA GGT TTG CCT AGA AG
38.	PLC 57	F: GGA AGT GAT TGT GGT TTT TAA TCA<br>R: ATT GCT CAT TCC CAC CAA AG
39.	PLC 58	F: TGG AAG AAA GAG AAG GGC AA<br>R: CAC AGC TAC CAA AAA TCA GTT CC
40.	PLC 59	F: TTG TTT AGC TGG TGT GGT TTT C<br>R: CTA CAG CAC GTT TGC AAG GA
41.	PLC 60	F: TGC TTG GAC CCT AAA TTT GC<br>R: AAG AAA AGG GCA ACC ACT GA
42.	PLC 61	F: ACT AGG AAA GGA AAA CGG CG<br>R: GAG TGA CAC GTG AAT GGT GG
43.	PLC 62	F: GCA AAG AAC AAG AAT AAC GTG G<br>R: CAA ACC GAA GAA TAA GAG AGG G
44.	PLC 63	F: TTG ATG GCT ATG GGA GTG GT<br>R: TGG TCC CAA CAA AAT ACC AA
45.	PLC 64	F: CAA ACT CTT CAC CGA CAC GC<br>R: AAC GAG GGT TAG GAT GAG AAG C
46.	PLC 65	F: TGT TGC AAT GCT TTT AGC CT<br>R: CAG AAG CTT TTC GGT GTT CC
47.	PLC 66	F: ATT TGG AGC AAA GAT GCA GG<br>R: GGA TCG ACC TCC AAT CAA GA
48.	PLC 67	F: GCA TAA TCA GTT TGT TTT TGC G<br>R: TTC TGC AAA AGC TTC TGG GT
49.	PLC 68	F: AAA AAG AGG CCA TCA TGT TCA<br>R: CAG CAG TGA CGG CAA TTT TA
50.	PLC 69	F: CGC TCT ACC AAC AGC ATA A<br>R: GAG GTC TCT TTT GTT CTT CAC T
51.	PLC 70	F: CAT CTC TTC GTG GCG TAA T<br>R: AGC AAA CAA CAG CAC ACA TA
52.	PLC 71	F: AGT GAG CAA GGA ATA AAA CG<br>R: GAG TAG CAA GGA AAG TGA AAA C
53.	PLC 72	F: TAT GAT GAA AGC CAG GAC A<br>R: GAC TGC ACA ATC TTA AAC ACC
54.	PLC 73	F: GAA AGG AAA GGT TTT AGC TG<br>R: CTT TGA TTG AGG TAA GAG CA

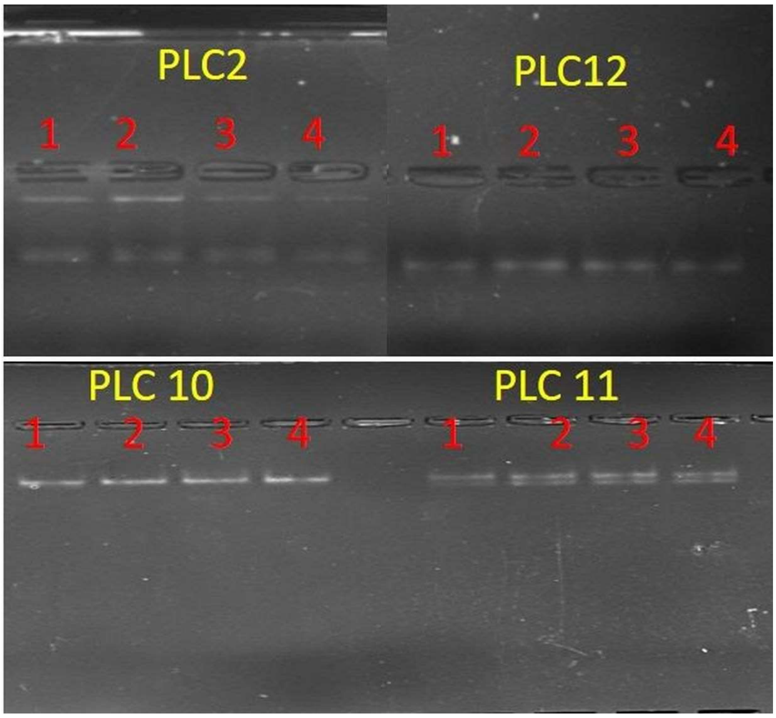

Figure S1. A representative picture showing the amplification details in four lentil samples (Differing for the seed coat color) using SSR markers. Where, PLC2, 12, 10, and 11 are the primers and 1: L4717, 2: brown seed-coated, 3: Black seed-coated, 4: Spotted seed-coated (2-4 are obtained from L4717-NM genotype expressing mixed phenotype).
